# Vimentin promotes collective cell migration through collagen networks via increased matrix remodeling and spheroid fluidity

**DOI:** 10.1038/s42003-026-10506-3

**Published:** 2026-06-18

**Authors:** Minh Tri Ho Thanh, Arun Poudel, Shabeeb Ameen, Bobby Carroll, M. Wu, Pranav Soman, Tao Zhang, J. M. Schwarz, Alison E. Patteson

**Affiliations:** 1 Physics Department, Syracuse University, Syracuse, NY USA; 2 BioInspired Institute, Syracuse University, Syracuse, NY USA; 3 Biomedical and Chemical Engineering Department, Syracuse University, Syracuse, NY USA; 4 Department of Biological and Environmental Engineering, Cornell University, Ithaca, NY USA; 5 Department of Polymer Science and Engineering, Shanghai Jiao Tong University, Shanghai, China; 6 Indian Creek Farm, Ithaca, NY USA

**Keywords:** Cellular motility, Collective cell migration

## Abstract

Intermediate filaments (IF) are diverse and cell-type specific. The IF protein vimentin, expressed in mesenchymal cells and different cancer cells, is functionally associated with cell migration through fibrous tissues. Vimentin increases cell elongation needed for migration, yet also acts as a cytoskeletal cage that hinders cells squeezing through small spaces. To determine how vimentin facilitates cell migration through the extracellular matrix (ECM) and around neighboring cells in tissues, we examine the collective invasion of cell spheroids embedded in collagen networks. Unlike single-cell migration in collagen networks, the collective invasion of cells through the collagen network is increased by vimentin for both mouse embryonic fibroblasts (MEF) and co-cultures of MEF with MDA-MB-231 breast cancer cells. Using multiple experimental systems, we show that vimentin increases spheroid contractility and that vimentin-mediated collective cell expansion depends on matrix metalloproteinases (MMP), which degrade collagen networks. In addition, through advanced imaging and a computational 3D cell vertex model, we find that vimentin enhances the elongation of cells in spheroids embedded in collagen, indicating increased spheroid fluidity and active collagen contraction. Altogether, these results reveal new insights on vimentin’s effects in enhancing collective cell migration in 3D matrix environments through collagen remodeling and tissue fluidity.

## Abstract (web summary)

Vimentin intermediate filaments mediate collective cell expansion of spheroids embedded in collagen gel via enhanced collagen remodeling and elongation of cells in spheroids, indicating increased spheroid fluidity and collagen contraction.

## Introduction

In vivo cell migration is a complex, multi-step process that involves interactions between different cell types and the extracellular matrix (ECM). Cell migration in fibrous tissues is aided by cellular secretion of matrix metalloproteinases (MMPs) to degrade the ECM and as well as generation of cellular forces that pull on the ECM network. The primary cells responsible for remodeling the extracellular matrix are fibroblasts, which help maintain homeostatic conditions and which will also migrate to sites of injury to rebuild the tissue^1,2^. Fibroblasts are abundant in tumor tissue and can be involved in promoting the invasion of cancer cells into surrounding ECM^3,4^.

Cell migration critically relies on the cell’s cytoskeletal networks to polarize the cell and generate forces. Intermediate filaments are abundant cytoskeletal polymers, largely recognized for their functional diversity and as a physical scaffold that gives the cell mechanical strength^5,6^ and mediates cell-cell signaling^7,8^. Mesenchymal cells, such as fibroblasts, express the type III intermediate filament protein vimentin. Thus, vimentin is a widely used marker of the epithelial-to-mesenchymal transition, in which cohesive epithelial cells lose their cell-cell contacts and transition to a polarized, motile mesenchymal phenotype^9^. Vimentin is important for wound healing^10^, but it is also highly expressed in metastatic cancers and tumors, correlating with poor patient prognosis^11,12^.

In many ways, the vimentin phenotype has been largely elusive. In 1994, the vimentin-null mouse was reported to develop and reproduce without an obvious phenotype^13^, but in 2000, it was found that the vimentin-null mouse had impaired wound healing abilities^14^. Since then, there have been at least 39 phenotypes revealed by the vimentin-null mouse linked to a variety of functions, including tissue remodeling, vascular functions, fat accumulation, tumorigenesis, and viral and bacterial infections^15^. Recent studies have hinted at mechanistic insight into vimentin’s role in cell migration and matrix remodeling relevant to in vivo conditions. Berr et al. and Etienne-Manneville et al.^16,17^ have shown that vimentin-expressing cell spheroids show increased cell expansion in ECM networks. Despite these observations, the main functions of vimentin in cell migration through ECM environments remains poorly understood, as at the single cell level, vimentin decreases 3D cell migration speeds^18^ and can also reduce single-cell contractility^19–21^. Further, a recent study by Ostrowska-Podhorodecka et al.^22^ showed that vimentin impacts the transport of the membrane type 1 (MT1)-MMP, whose role in degrading collagen is essential for collagen remodeling and which may be critically important for making room for collective cell migration through collagen networks.

In this manuscript, we use a three-dimensional model of cell spheroids embedded in collagen I matrices to probe the role of vimentin in collective cell migration. Collagen I is the main component of the ECM in connective tissues and solid tumors^23^, and gels formed by collagen I are a common model system for the tissue microenvironment^24,25^. Spheroids comprised of fibroblasts mimic physiological conditions in which fibroblasts detach from connective tissues, during inflammation, wound healing, and cancer cell migration^26^. To investigate the effects of vimentin on collective cell migration in 3D ECM environments, we developed and examined spheroids of wild-type and vimentin-null mouse embryonic fibroblasts embedded in collagen I matrices with and without co-culturing with MDA-MB-231 breast cancer cells. We find that vimentin significantly promotes the collective migration through collagen for both fibroblast spheroids and spheroids of mixed populations of fibroblast and cancer cell spheroids. To understand these effects, we further examine the role of vimentin network structure, spheroid contractility, and collagen degradation activity on vimentin-mediated collective cell migration. Our results show that an intact vimentin cytoskeleton is a significant contributor to collective cell invasion in collagen network and suggest new mechanisms by which vimentin mediates collective cell spreading and motility in real tissue.

## Results

### Vimentin enhances collective cell migration through collagen networks

**Fig. 1:**
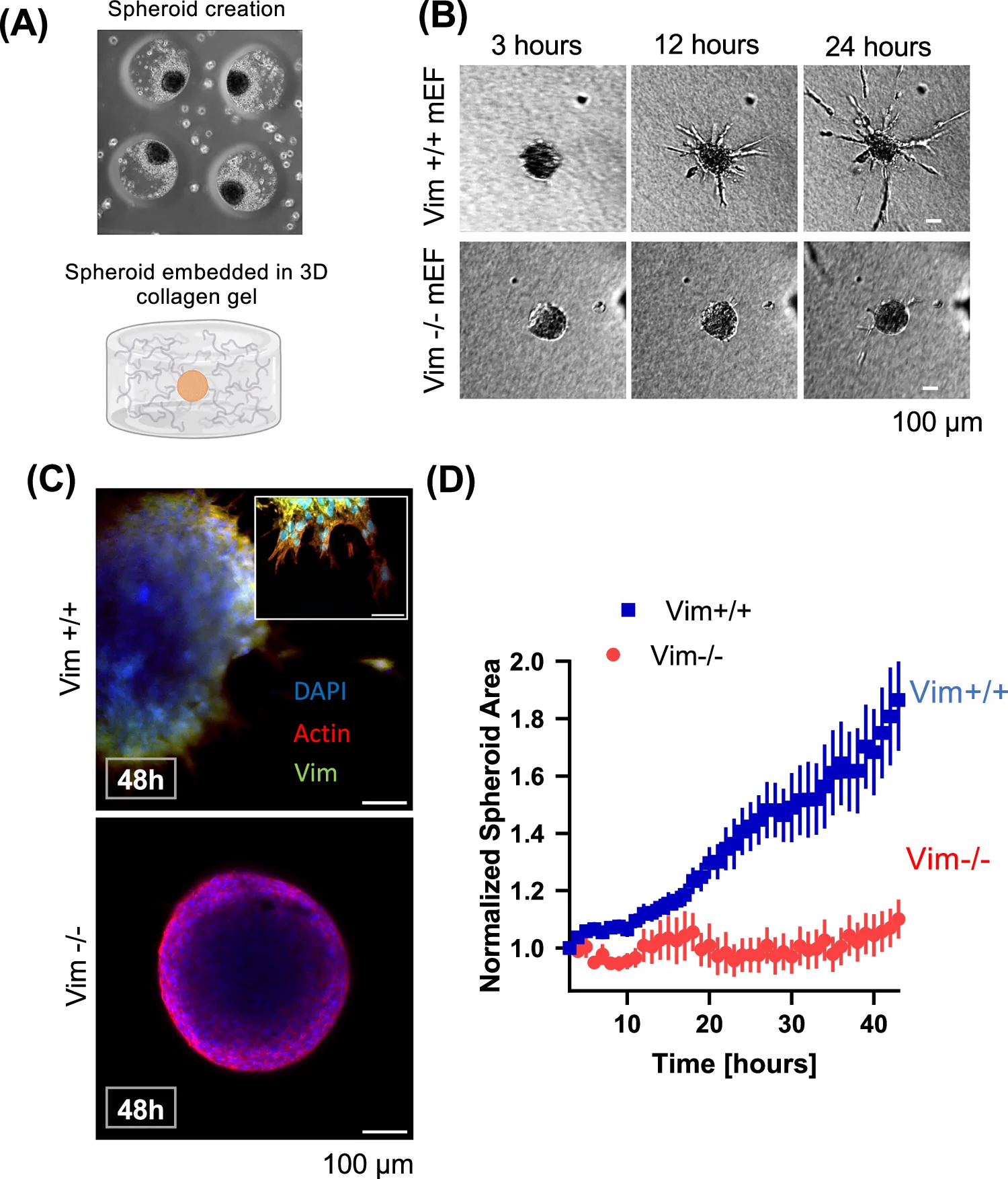
Vimentin promotes collective cell spheroid migration through collagen I networks. **A** Spheroids formation in fabricated microwell (r = 200 μm). Spheroids are then embedded in a collagen gel (1.5 mg/ml), as shown schematically. (Schematic made with BioRender. Patteson, A. (2026) https://BioRender.com/4esj8h7). **B** Representative timelapse images of Vim + /+ mEF and Vim–/– mEF spheroids spreading out in the 3D collagen gel. **C** Confocal images of Vim + /+ and Vim–/– spheroid in collagen gel at the 24-h time point (Blue: Nucleus, Green: Vimentin, Red: Actin). Inset: Zoomed images of the cells at the protrusion area. **D** Normalized spheroid area of Vim + /+ and Vim–/– spheroids over 48 h. Vim + /+ spheroids expand into collagen gel 60% more than Vim–/– spheroids. (n ≥ 4 independent experiments, N ≥ 21 spheroids for each condition, error bars represent SEM).

To investigate the role of vimentin in 3D collective cell migration, we developed fibroblast spheroids comprised of either wild-type or vimentin-null mEF. Spheroids were generated by culturing suspended cells in non-adhesive wells for 48 h (Fig. 1A, S1, Methods), creating spheroids 100–200 μm in diameter. Spheroids were then collected and embedded in collagen I matrices (collagen concentration = 1.5 mg/mL).

Representative time-lapse images of spheroid invasion are shown in Fig. 1B. Wild-type mEF spheroids display characteristic features of collective cell expansion in ECM network, in particular detachment of individual cells into the network as well as collective streams of cells radiating out from the core of the spheroid. The vimentin-null spheroids display very different behavior: cell migration into the surrounding network is strongly diminished. At 8 h, some cell protrusions in the vimentin-null spheroid can be seen invading the network. These protrusions are dynamic, and many collapse back toward the spheroid core (SI Movies 1, 2). A few vimentin-null cells can be seen to form stable protrusions into the network or detach from the spheroid and into the network by 48 h, though markedly less than the wild-type mEF.

To quantify the expansion, we identified and tracked the boundary of the cell spheroid using a custom image analysis thresholding code (“Methods”). We measured the mean projected area of spheroids, which includes the core of the spheroid and connected cell protrusions but not detached cells over time (“Methods”). Figure 1D shows the mean projected area of the spheroids normalized by their initial area. Vimentin enhanced the spreading of the cell spheroid by 80% compared to the cells without vimentin (*p* < 0.01). Overall, the results in Fig. 1 indicate that vimentin regulates 3D cell migration through collagen networks and promotes the expansion of fibroblast spheroids through an ECM network.

### Vimentin increases collagen network remodeling and spheroid contractility

**Fig. 2:**
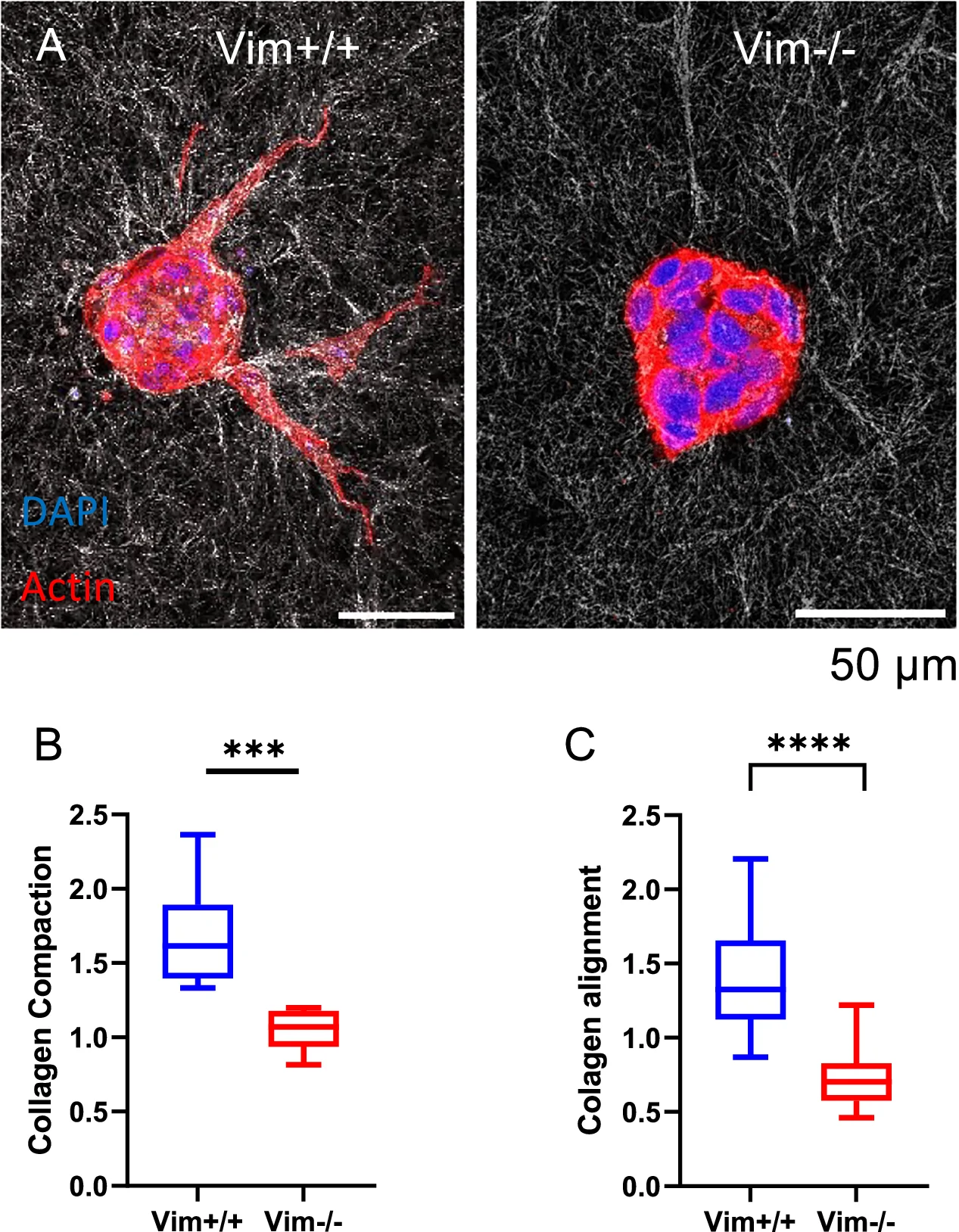
Vimentin increases spheroid collagen remodeling. **A** Confocal reflectance image of Vim + /+ and Vim–/– spheroid in collagen gel (Blue: DAPI, Red: Actin, White: Collagen). **B** Collagen compaction and **C** collagen alignment analysis of Vim + /+ and Vim–/– spheroids. Vim + /+ spheroids significantly compact, and align the network compared to Vim–/– spheroids (*n* ≥ 3 independent experiments, *N* ≥ 11 spheroids for compaction and *N* ≥ 24 spheroids for alignment for each group).

**Fig. 3:**
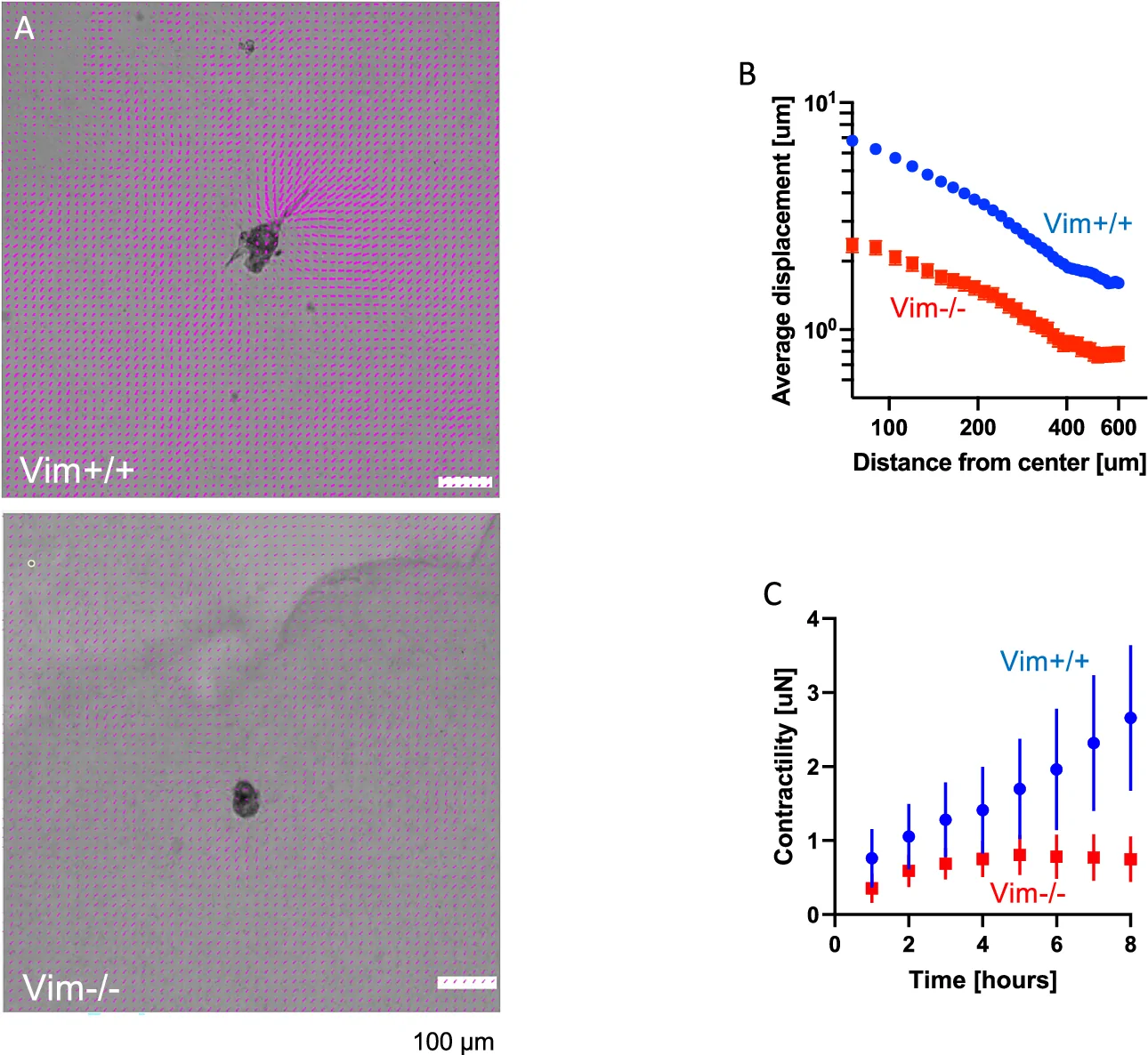
Vimentin enhances spheroid contractility. **A** Displacement field snapshot of a Vim + /+ and Vim–/– spheroid. **B** Average displacement as a function of distance away from the spheroid center (log-log plot). **C** Effective contractility of Vim + /+ and Vim–/– spheroids over 8 h (pre-invasion). Vim + /+ spheroids contract collagen gel more and further than Vim–/–. (*n* ≥ 3 independent experiments, *N* ≥ 12 spheroids for each group, error bars represent SD).

Next, we quantified the remodeling of the collagen network using reflective confocal imaging and traction-force microscopy-based techniques, as shown Figs. 2, 3. Confocal reflectance microscopy uses back-scattered light to form an image and is commonly used for imaging collagen networks simultaneously with fluorescence from cells^27^. Figure 2 shows representative images of the spheroids after 48 h in the collagen gel. The wild-type spheroids show a collagen-pulling behavior with fibers radiating out from the spheroid, consistent with the remodeling of collagen associated with spheroid expansion through ECM networks^28–30^. In contrast, a lack of radial fibers is observed in the vimentin-null spheroid. To analyze the collagen remodeling, we computed the compaction of the collagen network around the spheroids (Fig. S2, “Methods”), which showed an increase in collagen compaction in the vimentin-containing spheroids by more than 50% (*p* < 0.05). We also quantified the alignment of collagen fibrils around the spheroids (Fig. S2, Methods) and found two times increase in collagen alignment in wild-type compared to vimentin-null spheroids (*p* < 0.0001).

Contractility-based experiments were conducted by embedding the collagen networks with 2 μm fluorescence beads and performing time-lapse imaging of the collagen networks around the spheroids over time (SI Movies 3, 4). The videos show fluctuations of the tracer beads around the spheroids but a systematic net motion of the beads toward the spheroids over many hours, indicative of the generation of contractile pulling forces of the spheroid on the ECM network. To compare the wild-type and vimentin-null spheroids, we computed a time-aggregated (Fig. S7A) and time-averaged displacement field (Fig. 3). The displacement fields show a net inward contraction of the collagen toward the spheroids, and the magnitudes of collagen displacement are greater for the wild-type compared to vimentin-null spheroids. An interesting feature of the wild-type fibroblast spheroids is nonuniform regions of increased contraction that correlate with regions of outward invading strands of cells (Fig. 3A). The spatial differences in collagen displacements between the two spheroid types are highlighted by radially averaged displacement as a function of r from the center of the spheroid (Fig. 3B).

To connect the collagen deformation fields to forces generated by the spheroids, we use the finite element approach described in Mark et al.^31^ (“Methods”, Fig. S3). In this model, the spheroid is considered as a uniformly contractile sphere in a fiber-based network with mechanical properties and pore size parameters determined by experimental data. By fitting the experimentally measured collagen displacement data to the finite element results, we can extract an effective contractility that the spheroid exerts on the collagen network as a function of time. As the model assumes a spherical shape for contractility measurement, we quantified the effective contractility pre-invasion (up to 8 h). With this approach, we find that the effective contractility of the wild-type spheroids is significantly greater than the vimentin-null cell spheroids, as shown in Fig. 3C. In particular, the contractile force of the wild-type cell spheroids is approximately 2.7 μN compared to 0.75 μN for the vimentin-null spheroids. These data indicate that vimentin increases the contractility of collective cell spheroids in a soft 3D collagen network, consistent with the increased migration of the wild-type cells seen in Fig. 1.

### Vimentin-mediated collective cell migration depends on physical vimentin networks

**Fig. 4:**
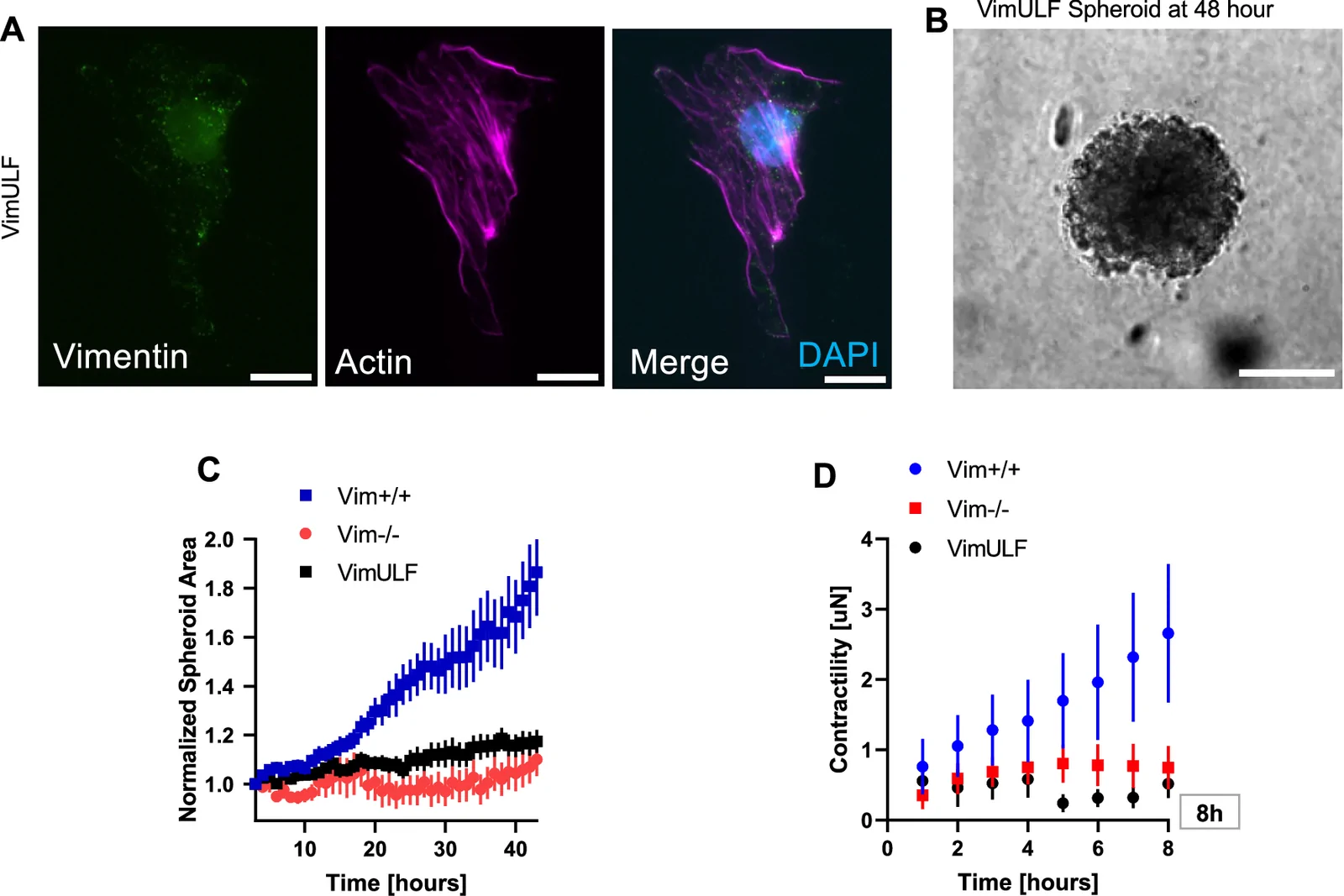
Vimentin filamentous networks are needed for collective expansion of fibroblasts in collagen. **A** Immunofluorescence images of Vimentin-ULF fibroblasts (Blue: DAPI, Green: Vimentin, Magenta: Actin. Scale bar: 10 µm. **B** Image of a spheroid made of VimULF fibroblasts embedded in Collagen gel (1.5 mg/ml) at 48 h. Scale bar: 50 µm. **C** Normalized Spheroid Area and **D** effective contractility comparison between Vim + /+, Vim–/– and VimULF spheroids. VimULF cells exhibit similar phenotype to Vim–/– spheroids. (*n* ≥ 3, *N* ≥ 14, SEM error bars for spheroid area; n ≥ 3 independent experiments, *N* ≥ 8 spheroids for each group, SD error bars for contractility data).

Our results thus far demonstrate that vimentin correlates with the invasiveness of fibroblasts through collagen networks. To investigate whether the physical structure of vimentin networks is essential for the observed collective cell migration, we next studied the “unit length filament” (ULF) vimentin mutant Y117L (Fig. 4A). The vimentin unit-length filament, consisting of approximately 8 vimentin tetramers and 60 nm long, is considered the smallest repeating unit in vimentin polymers^32^. These ULF mutants are prepared by expressing ULF vimentin in vimentin-null cells and thus retain vimentin’s signaling interactions within the cell, but its assembly into long filaments is blocked at the ULF stage^33^. Figure 4B shows that the knockout cells expressing ULF vimentin segments did not rescue the expansion behavior of wild-type cells. These ULF cells migrated poorly into the matrix and generated low contractility, similar to the vimentin-null spheroids. Figure 4C, D data suggest that intact vimentin networks are required for collective cell expansion in through collagen networks. Knocking down vimentin, which disrupts the VIF network, also resulted in impaired cell invasion, similar to vimentin-null and ULF cells, while rescuing vimentin in vimentin-null cells recovers the wild-type phenotype (Fig. S5).

### Vimentin-mediated migration is MMP-dependent

**Fig. 5:**
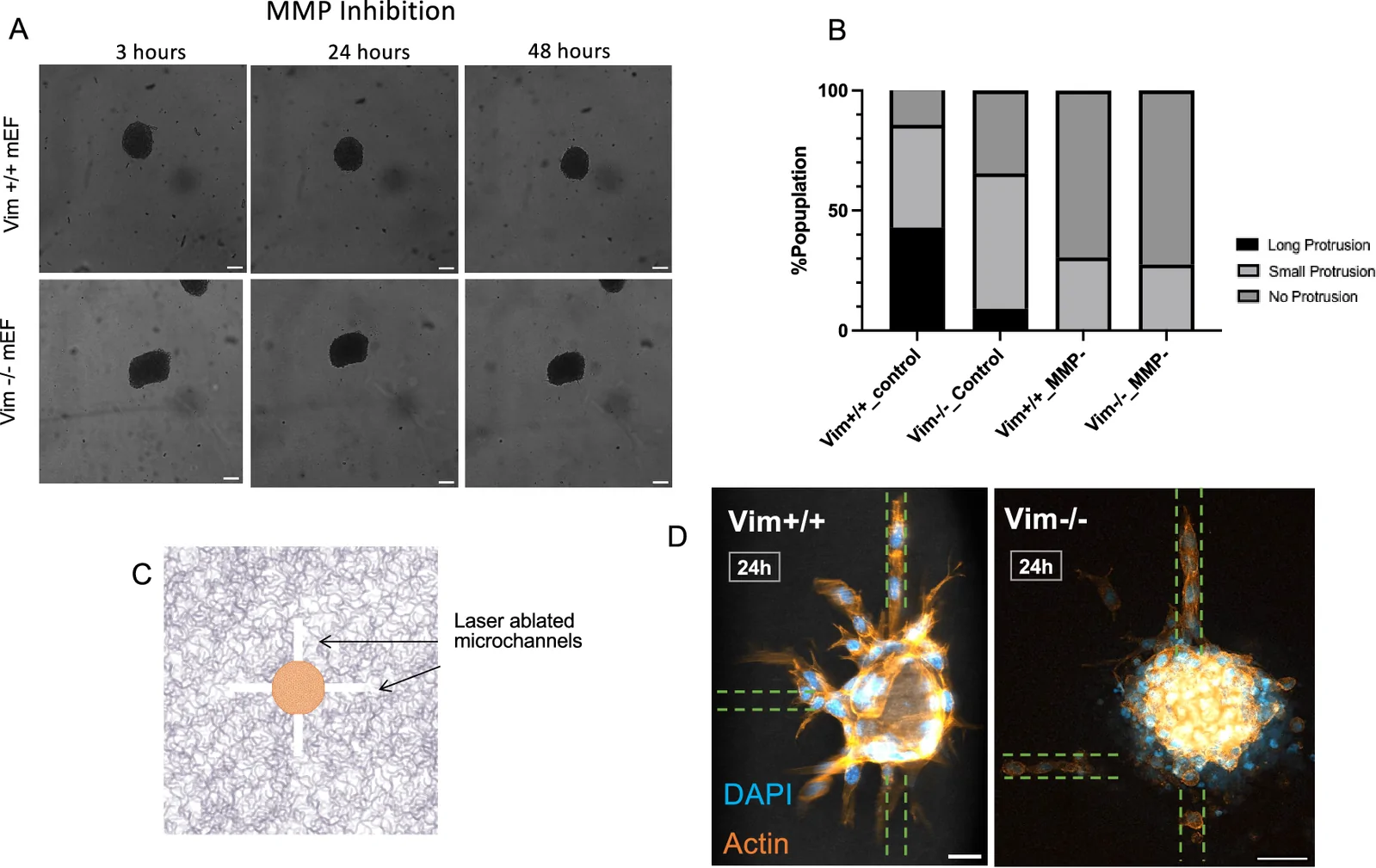
Fibroblast migration depends on collagen degradation. **A** Image of Vim + /+ and Vim–/– spheroids at 3, 24 and 48 h treated with GM6001 broad spectrum MMP inhibitor. Scale bar: 50 µm. **B** Quantitative analysis of spheroids population that shows protrusion. Long protrusion indicates strand invasion that is longer than 50 μm (approximately half of the spheroid radius). Small protrusion indicates that invasive morphology can be seen, but the strand length is smaller than the spheroid’s radius. MMP-inhibited spheroids are no longer capable of high invasion regardless of cell line. **C** Schematic of laser-ablated channels in collagen gel. (Schematic made with BioRender. Patteson, A. (2026) https://BioRender.com/c4lyo3f). **D** Fluorescence images of the Vim + /+ and Vim–/– spheroid in ablated collagen gel. Vim–/– spheroids are capable of invasion into the ablated channels. Scale bar: 50 µm. These experiments show that the invasive process is MMP-dependent and supported by vimentin.

Prior studies highlight that the migration rates of cells through collagen strongly depend on matrix metalloproteinases (MMPs), which degrade ECM fibers and create space for migration. To determine the role of MMP in the spheroid expansion, we treated the embedded spheroids with 25 µm of GM6001 broad-spectrum MMP inhibitor (Fig. 5A, “Methods”). The MMP inhibitor strongly suppressed the expansion of wild-type cells into the collagen network and decreased spheroid contractility (Fig. 5B). It strongly suppressed the formation of any long protrusions (> radius of the spheroid).

Based on these results, we hypothesized that the decreased migration of vimentin-null cells could come from alterations in collagen degradation. Further, the vimentin-null cells may be capable of migrating into the collagen network if the confinement from the matrix is alleviated. To test this scenario, we next performed laser-ablation experiments to generate microtracks in the collagen network around the cell spheroids, as shown in Fig. 5C. We found that the wild-type cells move out into the microtracks away from the spheroid (Fig. 5D), while also making their own tracks of protruding cells, as seen in the unablated collagen gels (Fig. 1). Interestingly, we also found that the vimentin-null cells made their way through the ablated microtracks. On average, 2–4 cells escaped approximately 100 μm in the microtracks for both cell types. The vimentin-null cells did not make their own protrusive stands in the unablated areas of collagen surrounding the spheroid. These data suggest that the confinement of the collagen matrix here is a major barrier for the vimentin-null cells’ motion and that these cells are able to move in collagen when that barrier is removed.

### 3D segmentation shows vimentin increases nuclear elongation in collagen-embedded spheroids

How does vimentin increase collagen cell contraction of the ECM network that is known to promote migration? The experiments thus far indicate vimentin-mediated migration is dependent on degradation of the collagen network, which could open up space for cells to better grip and pull-on collagen fibers. A possible parallel mechanism is related to the fluidization of the collective cell aggregate, spheroids, or tumors, which is connected to elongation of cells and increased migration^34–37^. In this context, the degree of fluidity of a collective cell aggregate refers to the ability of cells to rearrange and diffuse among their neighbors in the aggregate, allowing for motion and mechanical features akin to fluid flow. There has been much recent work on understanding this rigidity transition of different tissues, in other words, the transition from a stable solid-like structure to a fluid-like tissue, which is relevant to the collective motion of cellular aggregates in vivo. One finding that has been well described is that the distribution of cell shapes in epithelial-like tissues can predict where the aggregate is in relation to the rigidity transition, e.g. whether the aggregate will behave as a solid- or fluid-like material^38,39^. More elongated cell shapes, based on a cell-shape index, are associated with more fluid-like behavior. Recent work indicates a similar transition occurs in 3D spheroid aggregates^36,40^, and there is a close relation between cell shape and nuclear shape^41,42^.

**Fig. 6:**
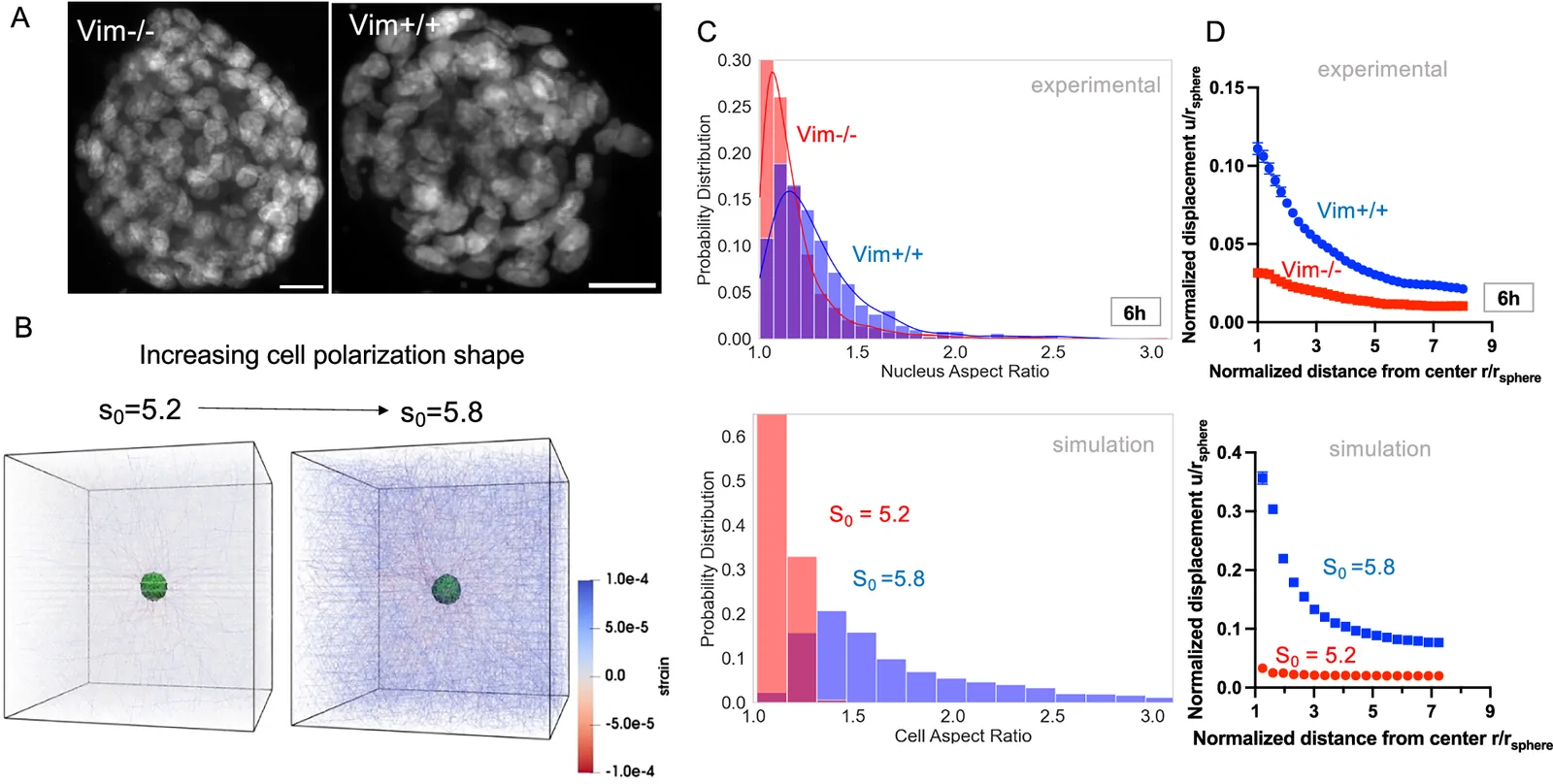
Vimentin increases spheroid fluidity, which is linked to collagen network contraction. **A** 3D confocal scan of cell nuclei in embedded spheroids for Vim–/– and Vim + /+ nuclei cells at 6 h (before significant invasion into the ECM has begun). Scale bar = 20 μm. **B** 3D vertex model of spheroids embedded in a cross-linked fiber network. The dimension shape factor index s_0_ characterizes elongation of the cell, which positively correlates with spheroid fluidity. Representative images are shown of simulated spheroids (s_0_ = 5.2 and s_0_ = 5.8) with collagen fibers color-coded by fiber strain from spheroid contraction forces. **C** Distribution of nucleus aspect ratio in the spheroids (top) and cell aspect ratio in the computational model (bottom). **D** Experimental (top) and simulated (bottom) collagen displacement as a function of normalized distance from the center of the spheroid. Both displacement and distance from the center is normalized against r_sphere_ – radius of the spheroid).

Motivated by these findings, we measured the distribution of 3D nuclear shapes in our wild-type and vimentin-null spheroids, which is technically more feasible than measuring 3D cell shapes in a spheroid. Nuclear shapes were assessed by running TWANG Segmentation code^43^ on confocal images of nuclei from embedded spheroids (Fig. 6A, S4A). We extracted the major and minor axis of the nuclei data to calculate nucleus aspect ratio (“Methods”). As shown in Fig. 6C, we found that the nuclei in the wild-type spheroid were significantly more elongated compared to the vimentin-null spheroid at 6 h (pre-invasion) post embedding spheroids into the collagen gel. The wild-type spheroids, in particular, exhibit more elongated nuclei, which tend to localize around the perimeter of spheroid, consistent with prior studies^36,40,44^. The wild-type distribution of nuclear shapes thus exhibits a maximum around 1.2, for the cells in the center of the spheroid, and a long tail at high nuclear elongation shapes for the boundary cells. This skewed distribution is also observed post-invasion (Fig. S4B): at 48 h, the vim + /+ distribution started to shift to the right, and more nuclei can be observed with aspect ratio of 1.8 to 2.3, with some highly elongated nuclei reaching aspect ratios of 2.8.

Taken together, these data point to an additional effect of vimentin on the collective cell expansion, namely vimentin increases nuclear elongation, a characteristic feature of increasing tissue fluidity. This connection exists at a pre-invasion stage (6 h), either independently or in parallel with degradation of the surrounding collagen that proceeds invasion.

### Increased tissue fluidity enhances collagen remodeling

To establish an interpretive framework for our results (Fig. 6), we invoked a novel 3D computational model of a cell spheroid embedded in a collagen network (SI)^37^ and compared the predictions of the model to our experimental results. As shown in Fig. 6B, the spheroid is comprised of a 3D vertex model, in which cells are represented as deformable polyhedrons that share faces with neighboring cells. Cells on the periphery of the spheroid are connected to a collagen matrix, which is well represented as a cross-linked network of fibers, and cells in the spheroid actively pull on the fibers connected to them. While prior literature work has developed models of contractile spheroids in collagen^31,45,46^, the 3D vertex spheroid model allows connections between individual 3D cell shapes, mechanics, motility, and the remodeling of the collagen network around the spheroid. Specifically, elongated cell shapes reflect higher cell motility, whereas less elongated, more spherical cell shapes indicate an arrested, less motile cell state^36,47,48^. Cell shape is assessed through *s*, a dimensionless shape index that depends on the cell target surface area A and cell volume V, defined as $s=A/{V}^{2/3}$, where V is the volume of the cell. Larger *s* indicates more elongated cell shapes. Finally, in addition to cells and collagen, we represent the connections between cells and fibers mediated by integrin complexes as active springs, acting as molecular clutches, bridging the face of a boundary cell and the nearest node in the fiber network to arrive at an embedded spheroid system^37^. A mathematical description of the model and the simulation parameters used are presented in the supplementary information.

Figure 6B shows computational pictures of spheroids embedded in collagen networks for spheroids with target cell shape indexes s_0_ of 5.2 (solid-like) and 5.8 (fluid-like). A mathematical description of the models and the simulation parameters used are presented in supplemental information. The choices of parameters, such as the fiber edge occupation probability *p* = 0.85, which optimally represents the 1.5 mg/ml collagen gel fiber density^37,48^, are explored in further detail in Tao Zhang et al. The data is shown for a time point sufficiently large enough to allow for both the cells and fibers to deform under the cell pulling forces to rearrange and reach a new steady state that differs from the initial state (SI). The pictures show that collagen remodeling and network strain significantly increase for the high target shape index s_0_ = 5.8, compared to s_0_ = 5.2. The remodeling of the fiber network is a consequence of a positive feedback mechanism between the fiber network and the spheroid. Deformations in the spheroid apply strains on the fiber network, which remodels accordingly. The remodeled fiber network, in turn, changes the profile of applied stress on the spheroid. Spheroids with greater fluidity are susceptible to larger shape deformations (via increased reconnection events for cells within the spheroid itself) under such variations in applied stress. Hence, fluid-like spheroids enhance the remodeling of the surrounding collagen. The coupling between the spheroid and the fiber network, which facilitates this positive feedback, is modeled using active linker springs, again, acting as molecular clutches.

To quantify the feedback, we measured the collagen concentration, collagen compaction (Fig. S4C) and collagen displacement as a function of the radial distance away from the center of the spheroid from our model. All three of these metrics—which quantify different aspects of collagen remodeling—increase for spheroids with increased cell elongation. Indeed, as shown in Fig. 6C, we observed that the simulated cell aspect ratio distribution closely resembles that of experimental nucleus aspect ratio distribution. These data indicate that more elongated cells, reflective of a more fluid-like spheroid, pull on and rearrange surrounding collagen to a greater extent than non-elongated cells, reflective of a more solid-like spheroid. This effect is the result of increased cell dynamics and cellular rearrangement that emerge over time in the fluid-like spheroid, allowing cells to increase their frequency of contact and pulling on the collagen surrounding the spheroid^35^.

This computational model captures the main features of the experimental data and connects cell shape with collagen remodeling. Namely, we find spheroids with more elongated cells—as indicated by the elongated nuclear shapes in wild-type cells—exhibit more collagen remodeling in the spheroid-collagen model presented here (Figs. 6D, S4C). Furthermore, for both cell types, the computational model corroborates the decrease in collagen remodeling with increasing distance from the spheroid. We note that quantitative discrepancies between the experimental and computational data here are expected, as we have not yet included all potentially relevant biological processes. For instance, cells in experiments actively remodel and degrade the collagen network over time, effectively reducing network stiffness and enabling larger displacements. The simulated displacements are therefore systematically smaller than those observed experimentally. In other words, we interpret the model predictions as providing a quantitative lower bound on matrix displacements in the absence of active biochemical remodeling. Nevertheless, these analyses were done on spheroid preinvasion, our data give rise to the intriguing hypothesis that vimentin increases collective cell migration by (at least in part) elongating cells and increasing tissue fluidity, which supports enhanced collagen remodeling at the collective cell level. Because the compaction and cell-induced stress of a collagen network feeds back into cell migration^49^, this effect further promotes the expansion of the cells into the collagen network, in addition to elongation and fluidization of the cells themselves. More broadly, our experimental and computational data supports the inference that a more fluidized spheroid (as indicated by elongated nuclear shape) more strongly rearranges a surrounding collagen network, which, to the best of our knowledge, has not been previously demonstrated in this detail and is an understudied aspect of spheroid expansion in collagen networks.

### Vimentin intermediate filaments aid collective escape of fibroblast-cancer cell co-cultures

**Fig. 7:**
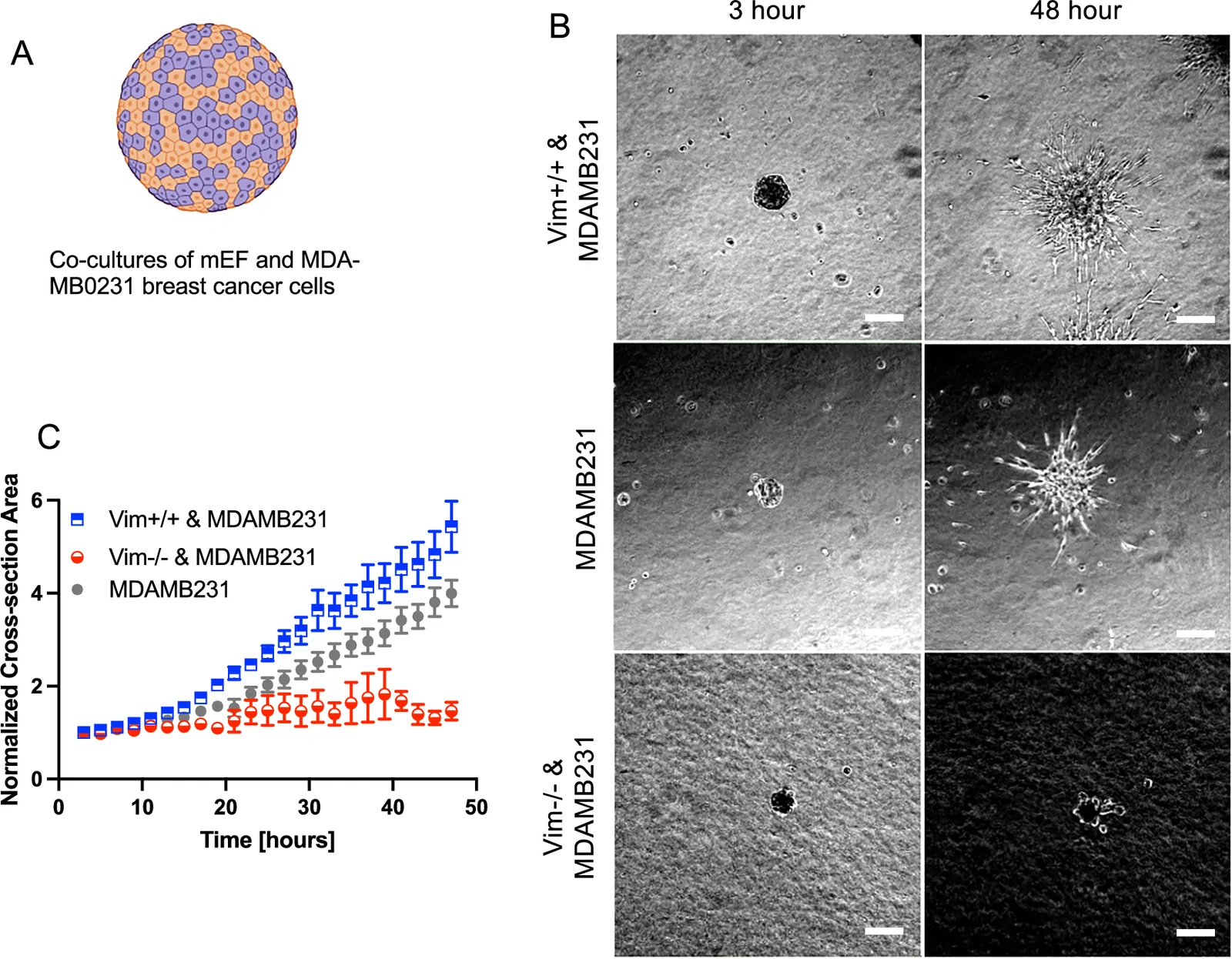
Vimentin-expressing fibroblasts promote collective migration of co-culture spheroids with breast cancer cells. **A** Schematic of co-culture spheroids and normalized spheroid area comparison of the co-culture spheroids and monoculture cancer spheroids over 48 h (Schematic made with BioRender. Patteson, A. (2026) https://BioRender.com/o4mpsxp). **B** Morphology of spheroids at 3 and 48 h for: Vim + /+ mEFs and MDAMB231, MDAMB231, and Vim–/– mEFs and MDAMB231. Scale bar: 50 µm. **C** Normalized spheroid area of Vim + /+ mEFs and MDAMB231, MDAMB231, and Vim–/– mEFs and MDAMB231 over 48 h. Co-culture with Vim + /+ mEFs enhances the invasiveness of the cancerous spheroid. (*n* = 3 independent experiments, *N* ≥ 9 spheroids for each group, error bars represent SEM).

The role of vimentin in enhancing collective migration of fibroblasts in collagen gels may have significant physiological consequences. For example, fibroblasts play an important role in growth and expansion of tumors into the surrounding soft matrix environments^3,4,50,51^. To test whether vimentin impacts fibroblast function in cancer cell migration, we conducted experiments with fibroblast-cancer cell co-cultures. Specifically, we generated spheroids comprised of both mEF and the breast cancer cells MDA-MB-231 (Fig. 7A, “Methods”) at a ratio of 2:1 consistent with prior co-culture models^52,53^. We found that the presence of vimentin in the fibroblasts of the co-cultures had a significant impact on the spheroid expansion. Figure 7B shows representative images of spheroids comprised of (i) MDA-MD-231 cells alone, (ii) wild-type mEF and MDA-MB-231 cells combined, and (iii) vimentin-null mEF and MDA-MB-231 cells. Notably, the wild-type mEF and MDA-MB-231 cells showed increased expansion compared to the MDA-MB-231, pointing to the role of fibroblasts in aiding cancer cell migration. In striking contrast, we found that expansion of co-cultures of vimentin-null mEF and MDA-MB-231 was almost entirely suppressed. The co-cultures of vimentin-null mEF and MDA-MB-231 results are a function of the proportion of vimentin-null cells to MDA-MB-231s (SI). Overall, the data in Fig. 7C indicates that vimentin has a strong impact on the function role of fibroblasts in co-culture cancer cell migration, which could have important applications for treating cancer by targeting vimentin.

## Discussion

Our results demonstrate that the vimentin cytoskeleton plays an important role in driving collective cell migration through ECM networks, as indicated by spheroids comprised of both fibroblasts alone and fibroblast-cancer cell co-cultures. This increased cell migration correlates with increased spheroid contractility, protease-mediated collagen degradation, and spheroid fluidity. Further, disrupting the assembly of filamentous vimentin networks suppresses collective cell migration through collagen, indicating the presence of filamentous vimentin is necessary for collective fibroblast migration through ECM networks. The differing effect of vimentin on single-cell versus collective-cell migration highlights the influence of cell-cell contacts and proteolytic activity during collective migration through fibrous networks.

There are several aspects of our findings that could be considered surprising for at least two reasons based on prior studies. First, recent work has shown vimentin diminishes actin stress fiber contractility by deactivation of RhoA through its guanine nucleotide exchange factor GEF-H1^20^. This study demonstrated loss of vimentin increased cell contractility. Here, we find that at the collective level, vimentin promotes spheroid contractility. This could be partly due to vimentin’s effects on cell contractility have been found to be matrix stiffness dependent^21^. Based on prior studies^21^, vimentin’s effect on cell contractility in a soft collagen network would promote the mechanical functions of the F-actin network that generates cell contraction. Second, vimentin has been shown to decrease cell speeds in confining 3D spaces. For individual cells in micro-channels and collagen networks, vimentin diminishes the speed of individual cells moving in micro-channels and collagen networks, which is opposite to its effect on collective fibroblast aggregates here^54^. For individual cells, the decrease in cell speed has been attributed to resistance of deforming the vimentin network to squeeze through small spaces, which confers protection to the cell nucleus^18^. Further, vimentin was found to reduce nuclear deformations and elongation under conditions of extreme external confinement, such as single cells squeezing through 3 μm collagen pores or cells are subject to uniaxial pressure^55^. Our work here suggests that, unlike single cells, spheroid-based cells can overcome this barrier to promote migration and spread out in 3D collagen networks at the collective level.

In light of the new data gathered here, we suggest a minimal model of collective cell migration in fibrous environments that captures the effects of vimentin. In this picture, vimentin has at least two different yet complementary effects, namely roles in collagen degradation and enhanced cell contractility via increased elongation.

First, as shown in Fig. 5A, collective fibroblast migration through collagen networks is MMP-dependent, at least for the collagen concentration used here, where the pore size is 2–5 µm: small compared to the cell size. The role of vimentin in collagen degradation has recently been reported. Using wild-type and vimentin-null mEFs, Ostrowska-Podhorodecka et al. showed that vimentin’s interaction with myosin-X was required for pericellular collagen degradation by MT1-MMP. In particular, vimentin deletion does not change expression of MT1-MMP in mEFs but lowers the secreted MT1-MMP and alters the localization of MT1-MMP in the plasma membrane. Absence of vimentin reduced MT1-MPP transport toward cellular extensions, down-regulated collagenolytic activity, and reduced collagen fiber alignment and cell invasion of the matrix^22^. Further, these effects required physical vimentin networks, not just soluble vimentin units, as shown using ULF cells, consistent with the reduced migration of ULF cells found here (Fig. 4). Likewise, van Bodegraven et al. found that intermediate filament depletion in glioblastoma cells reduces MT1-MMP expression while re-expression of VIM in IF-depleted GBM cells rescues MT1-MMP expression level. Inhibition of MT1-MMP reduces invasion in 3D Matrigel while expressing MT1-MMP rescues invasion in IF-depleted cells both in vitro and in vivo^17^. As greater collagen alignment promotes tumor invasion^56^, these effects could explain the reduced cell invasion seen in spheroids of mixed vimentin-null – MDA-MB-231 cells, in which some of the proteolytic-capable MDA-MB-231 are replaced with proteolytic-, alignment-deficient vimentin-null cells. Further, as shown in Fig. 5D, vimentin-null cells from the spheroids are capable of moving out into the matrix, once they can reach large enough channels, generated here by laser ablation. Vimentin has also been shown to enhance ECM degradation by regulating MT1-MMP via podosome dynamics and turnover in macrophages^57^. However, fibroblasts typically do not form podosomes^58^ unless they are under special treatment^59^ or plating on fluid substratum (where cell-matrix force generation is prohibited)^60^.

A second finding is the evidence that vimentin increases fluidization of the collective fibroblast spheroids, as shown by the increase in nuclear elongation in Fig. 6C. Vimentin is known to increase cell elongation through its interactions with microtubules and actomyosin networks^61–64^. Interestingly, we have found that when cells are geometrically constrained to elongated shapes on micropatterned substrates, vimentin increases the transmission of internal acto-myosin based forces to the cell nucleus, leading to larger deformations and elongations of the nucleus^65^. One possibility for vimentin’s differing effects on nuclear shape is that there are differences in how vimentin mediates passive outside-in applied forces versus active internally generated forces. Namely, vimentin may resist large external deformations of the cell from outside boundaries that decrease nuclear deformations; it may also assist in the transmission of internal actomyosin contractility that deforms the nucleus by interlinking to the actomyosin network and to the nuclear envelope through the LINC complex via nesprin-3.

Given the diversity of intermediate filament functions and the complexity of collective cell migration, our study on spheroid expansion makes some assumptions and simplifications. In particular, we have yet to explicitly account for possible changes in cell-cell and cell-matrix adhesions between our two cell types for several reasons. Fibroblasts are differentiated to lose cell-cell contacts and have very few cell-cell adhesive proteins^66,67^. We have immunofluorescently stained for N-cadherins in these cell lines and received very little fluorescence signal (Fig. S7C). Furthermore, N-cadherins have been shown to not colocalize with vimentin in rat embryonic fibroblasts^68^ and N-cadherin expression is the same in both wild-type and vimentin-null mouse dermal fibroblasts supernatant media^69^. Thus, we expect both cell types to have a strong preference for cell-matrix adhesive contacts over cell-cell contacts. Further, prior studies have shown that vimentin-null cells are capable of strong cell-matrix adhesions, particularly at high cell densities in collagen networks^70^. We have also examined focal adhesion proteins (paxillin) and cell area on soft collagen-coated polyacrylamide gel (500 Pa) and have found they are statistically the same between the wild-type and vimentin-null mEF, indicating no significant changes in cell-matrix adhesions (Fig. S7B). This result is different than previously found on collagen coated glass, where vimentin changes the distribution, clustering, and recruitment of focal adhesion complex proteins^71^. The difference between gel and glass is mainly due to the focal adhesions are much more matured when the cells are fully spread out on glass compared to very soft gel.

Another possible explanation for the increased in invasiveness of the wild-type spheroids might be due to changes in proliferation rate. However, we have no report of proliferation defect for these mEFs in cell culture condition. In wound healing assay, Eriksson’s group has found that vimentin affects the proliferation rate and survival rate starting on day 2^69^. However, our normalized spheroid area of wild-type and vimentin-null spheroids under MMP inhibition shows no significant difference (Fig. S5E). We have also observed a significant difference in cell migration after 24 h (Fig. 1), so the increased in invasiveness is not expected due to purely higher cell proliferation or survival.

One area of future exploration is to address the relative extent to which collagen degradation and spheroid collagen contractility work in parallel to coordinate collective migration. One open question is if, for instance, when collagen-degradation was rescued in vimentin-null cells and ULF cells, would they generate greater contractility forces and phenocopy the expansion of wild-type cells. Exploring a greater range of collagen concentrations in future studies will also help elucidate vimentin’s role in stiffness-dependent contractility in spheroids and the relative role of collagen degradation as a function of pore size^72^.

Finally, fibroblasts are known for their ability to remodel tissue and to increase the motion of cancer cells through various factors, including contraction of the ECM network and degradation of the ECM through MMPs^73^. Notably, Withaferin A (WFA), a drug targeting vimentin, has displayed promising antitumor and antiangiogenesis activity in animal models^74^ and is currently being evaluated in phase I/II clinical trials for combination therapy in recurrent ovarian cancer. Understanding the mechanisms by which cells collectively navigate and remodel their matrix environments will help identify how migration is altered during disease and strategies to mitigate it.

## Conclusions

Altogether, our experimental results motivate a physical mechanism in which vimentin-mediated collagen degradation is a prerequisite for collective fibroblast migration in collagen networks, and loss of vimentin-mediated collagen degradation is a limiting factor. In this sense, our results suggest vimentin’s role in collagen degradation is a predominant effect in inhibiting collective migration in ECM networks. However, a secondary effect of vimentin is the elongation of cells associated with the fluidization of the spheroids, which promotes collective cell expansion and may aid in collective spheroid contractility.

**Fig. 8:**
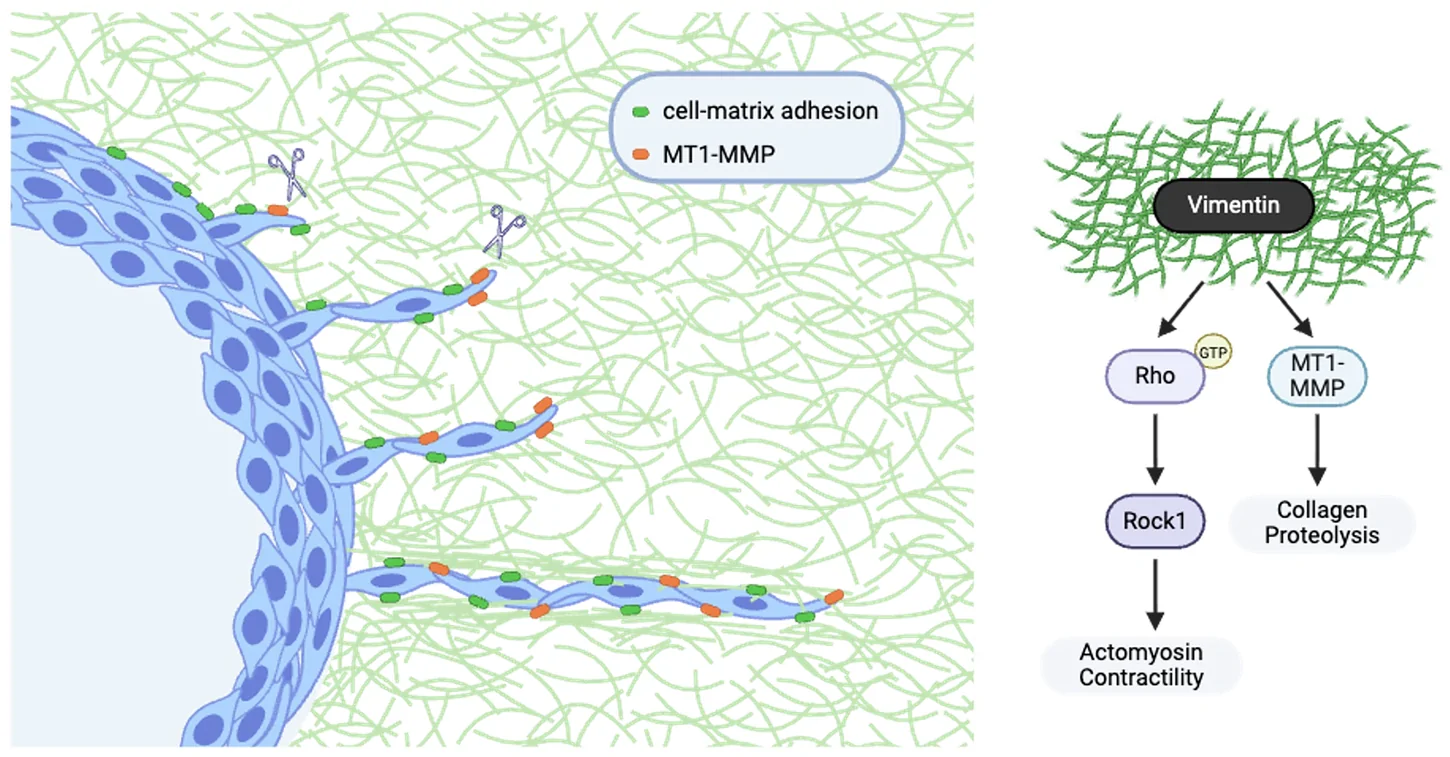
Summary schematic of vimentin’s effects on collective cell spheroid expansion in collagen networks. Vimentin-expressing mesenchymal cells form strong cell-matrix adhesion with collagen. As cells begin to form adhesions and protrusions into the network, vimentin promotes collagen proteolysis via MT1-MMPs that degrade the network to make space for new cells. In parallel, vimentin increases cell actomyosin contractility and elongation. These features aid remodeling and contraction of the collagen matrix to promote collective cell migration. Schematic made with BioRender. Patteson, A. (2026) https://BioRender.com/zwr8iw6.

In summary, we identify novel effects of vimentin on collective cell migration, summarized in Fig. 8. Collectively, vimentin impacts fibroblast spheroid matrix remodeling and spheroid fluidity, relevant to collective cell migration through soft connective tissue. Our studies highlight the importance of vimentin’s dual role in mechanical and non-mechanical signaling functions in recognizing its full impact and multifactored functions in cells and tissues. Our results also underline the significance of using spheroid-based versus single-cell-based migration assay. Future studies will be needed to determine the specific molecular mechanisms underlying vimentin’s role in collective migration and to decipher its influence at multiple different lengths and time scales associated with migration, ranging from ECM remodeling to cell shape and nuclear positioning.

## Materials and methods

### Cell Culture

Vimentin wild-type (Vim + /+) and vimentin knockout (Vim–/–) mouse embryonic fibroblasts (MEFs) were gifted by J. Eriksson from Abo Akademi. Vimentin unit length filament (VimULF) cells were gifted by the Vladimir Gelfand lab from Northwestern University. Vimentin Rescue mEFs were gifted by the Robert Goldman lab from Northwestern University. MDAMB231 human breast cancer cells were given by the Mingming Wu lab from Cornell University. For vimentin knockdown cells (VimKD), wild-type cells were trypsinized and plated on 6-well plates to about 70% confluence. The cells were then transfected with Dharmacon SMARTpool ONTARGETplus vimentin siRNAs plus transfection reagents or with non-targeting pool siRNA (control), using the manufacturer’s protocol (https://horizondiscovery.com/-/media/Files/Horizon/resources/Protocols/dharmafect-rna-transfection-quick-protocol.pdf). All cells were cultured in Dulbecco’s Modified Eagle’s Medium + 4.5 g/L glucose + 2nM L-glutamine + sodium pyruvate (DMEM, Gibco) and supplemented with 10% fetal bovine serum (FBS, Hyclone), 1% non-essential amino acid (Fisher Scientific), 1% penicillin/streptomycin (Fisher Scientific) and 25 nM of HEPES (Fisher Scientific). Cell cultures were maintained at 37°C and 5% CO2. Cells were passaged at confluency 70-80% and medium was replenished every 3 days.

### Spheroid culture

To create the spheroid invasion assay (Fig. S1), we first made microwells by adapting the PRIMO (Alvéole) spheroid protocol (https://www.alveolelab.com/resources-support-center/application-notes/). In brief, a 1% agar spin-coated glass slide was glued to the bottom of a 35 mm petri dish to create a non-adherent surface. Next PDMS stencils were assembled on top of the coated glass surface to create a hydrogel PDMS chamber. Then 50% 4-Arm-PEG-Acryl in PLPP Classic (PRIMO) solution was injected into the PDMS chamber. Next, the solution was exposed to UV light with the PRIMO device using Leonardo software with the appropriate photomask (well radius = 200 µm, well height = 250 µm, 45mJ/mm^2^ for 20 s). After the polymerization, the stencils were then removed and the microwells were washed under PBS 3x, exposed under UV light for 15 min and then incubated in cell media for 20 min. Finally, cells were plated at high density 80,000 cells/dish in DMEM/F12 (10% FBS, 1x Pen/Strep) to ensure the cells would get into the microwells instead of rolling off to the empty area. Cell aggregates were incubated at 37 °C with 5% CO_2_ for 2 days before harvesting.

### Collagen gel preparation

Collagen gels were prepared using a slightly modified protocol from Ibidi® (https://ibidi.com/img/cms/support/AN/AN26_CollagenI_protocols.pdf). Collagen type I, rat tail 5 mg/ml (Ibidi) were diluted on ice to 1.5 mg/ml with 6.67% of 5x DMEM (Fisher scientific), 10% FBS in 1x DMEM. The pH was adjusted to 7.4 using 1 M NaOH and sodium bicarbonate. Cell aggregates were added to the collagen solution before it solidified. The collagen solution is then pipetted on 1% agarose coated 24-well flat bottom plates (Corning) and let to polymerize for 40 min in an incubator at 37 °C, 5% CO_2_ and 100% humidity. DMEM complete media was added on top of the fully polymerized collagen gel.

### Characterization of spheroid-generated collagen network changes

Collagen compaction (Fig. S2A) was found with method previously used in ref. ^75^. In brief, the mean intensity of the confocal reflectance image of collagen fibrils in fixed area around the core spheroid was computed and then normalized against the mean intensity of the collagen fibrils at the corners (far away from the spheroid).

For measuring collagen alignment index (Fig. S2B), we utilized a method that has been done previously in ref. ^75^ with some modification due to strong scatter signal from the core of the spheroid. Nine sub regions surrounding the core spheroid of the confocal reflectance image of the collagen fibrils are cropped and transformed with 2D Fast Fourier Transform (MATLAB). The transformed image was then replotted as a function of radial sum of the intensity as a function of angle. The alignment index was defined based on higher pixel intensities in a specific angle, which was related to the orientation of collagen fibers in the corresponding direction. Quantification was done by calculation of the area under the intensity curve within ± 10 degrees of the peak using a self-written MATLAB script.

### Morphological spheroid quantification

After 3 h inside the incubator, spheroids in collagen gel are imaged for 48 h with phase-contrast widefield using a Plan Fluor 10× objective on Nikon Eclipse Ti inverted microscope equipped with an Andor Technologies iXon em+ EMCCD camera (Andor Technologies). Aggregates were maintained at 37 °C and 5% CO2 using a Tokai Hit (Tokai-Hit) stage top incubator. Cross-sectional area of the spheroid (core and connecting strands) was preprocessed with custom MATLAB code and traced using ImageJ.

### Nucleus segmentation

Segmentation was done using the segmentation pipeline XPIWIT which is a XML pipeline wrapper for ITK segmentation software^76^. The software package is available at https://bitbucket.org/jstegmaier/xpiwit/downloads/ and it has been verified to work very well on nuclear segmentation^77^. Raw DAPI channel of the spheroid confocal images were obtained and used as an image input for the software. The XML pipeline chosen was the default TWANGSegmentation provided (Fig. S4A). Custom MATLAB script was used to generate input file and PowerShell script to batch run XPIWIT for each confocal image stack. Output of XPIWIT was then queried into Microsoft Excel Data Model (Data Analysis Plugin) for data cleanup: removing duplicates, filter out nucleus volume that are too small (image artifact). Nucleus aspect ratio is calculated from extracted major and minor axis and plotted with Jupyter Notebook (Python). Statistic comparison between population was done using MATLAB.

### Effective contractility

FluoroSphere (2 µm) stock solution (ThermoFisher) was added to the collagen gel mixture (1.5 mg/ml) at 1:50 ratio. Spheroids were prepared as previously described and transferred into collagen gel mixture with fluorescent beads. Collagen gel was then polymerized in the incubator for 1 h and before cell media was added on top. After 3 h, spheroid was then mounted and imaged every 15 min for 48 h using Plan Fluor 10× objective on Nikon Eclipse Ti inverted microscope equipped with an Andor Technologies iXon em+ EMCCD camera (Andor Technologies) and Lambda LS (Sutter Instrumentation Company) for fluorescent excitation. Sample were maintained at 37 °C and 5% CO2 using a Tokai Hit (Tokai-Hit) stage top incubator.

Effective contractility was calculated using the jointforces package provided by Christoph et al. at https://github.com/christophmark/jointforces. In brief, beads displacement was computed using OpenPIV (https://openpiv.readthedocs.io/en/latest/) provided via jointforces package. The material simulation was perform using saenopy package (included in jointforce) which is a semi-affine fibers material model. The material model (not affine material) assumes the biopolymer network as a mechanical continuum with some affine properties, which is approximated by a mean-field description. Individual fibers are not simulated; the model uses a mean-field approximation to capture their collective mechanical effect^78^. The fibers are modeled to show different behavior in three strain regimes: under compression, fibers buckle showing exponential decay of the stiffness; under small strains, fibers are under linear stiffness regime; and for larger strain, fibers exhibit strain stiffening with exponential increase of the stiffness. The energy function *w”(ε)* is defined as:

$${{\rm{w}}}{{\hbox{''}}}({{\rm{\varepsilon }}})={k}_{0} \cdot \left\{\begin{array}{cc}{e}^{\frac{\varepsilon }{{d}_{0}}} & \hskip 5mm {if}-1\le \varepsilon < 0 \,({buckling}) \\ 1 & {if}\,0 \le \varepsilon < {\varepsilon }_{s}\,({linear})\\ {e}^{\frac{\varepsilon -{\varepsilon }_{s}}{{d}_{s}}} & {if}\,{\varepsilon }_{s}\le \varepsilon \,({stiffening})\end{array}\right.$$

where the stiffness is denoted as *k*_*0*_, the buckling exponent as *d*_*0*_, the stiffening threshold as *ε*_*s*_ and the stiffening exponent as *d*_*s*_.

Effective contractility was then finally reconstructed using PIV result output and material simulation lookup table. 1.5 mg/ml collagen lookup table was created using jointforce material simulation with parameters found from collagen rheology characterization (Fig. S3E): κ_0_ = 447 Pa, ε_s_ = 0.0075, d_s_ = 0.033, d_0_ = 0.0008.

### Laser-ablated channels

Laser ablation of microchannels in cell encapsulated collagen ECM was performed using a femtosecond (fs) laser platform, which combined a Ti:Sapphire fs laser (Coherent, Chameleon, USA) with a Zeiss Microscope (Observer Z1, Germany). In this setup, 800 nm fs-laser beam with a repetition rate of 80 MHz was focused into the crosslinked collagen using a water immersion objective (40, NA = 0.8, Leica). Visual basic code was employed to control the movement of microscope stage and the shutter, allowing for controlled scanning of laser focus within the collagen to ablate 2D microchannels. The laser dosage needed for ablation of collagen concentration was determined by varying laser power and scanning speed of the laser focus. Specifically, a laser power of 1.2 W, measured just before the objective, and scanning speed of 50 μm/sec was used to ablate microchannels.

### Statistics

Data is presented as mean ± standard error of the mean (SEM). Statistical analyses were performed using GraphPad Prism 9 (GraphPad Software, Inc.) unless stated. Comparison between Vim + /+, Vim–/– and VimULF are done using Welch ANOVA test (multiple comparison). Denotations: **** *p* < 0.0001, *** *p* < 0.001, ** *p* < 0.01, * *p* < 0.05. n denotes number of experiments and N denotes sample population of a phenotype. Error bars are plotted as standard error of the mean (SEM) or standard deviation (SD) as indicated in each plot.

### Reporting summary

Further information on research design is available in the Nature Portfolio Reporting Summary linked to this article.

## Supplementary information

Supplemental Information

Description of Additional Supplementary files

SupplementaryData

Supplementary Movie 1

Supplementary Movie 2

Supplementary Movie 3

Supplementary Movie 4

Reporting Summary

Transparent Peer Review file

## Data Availability

All source data used in figures is available in Supplemental Data. Any remaining data that support the findings of this study are available from the corresponding author upon reasonable request.

## Code availability

Simulation Code is available on GitHub: github.com/ZhangTao-SJTU/tvm/tree/3DVM-ECM. Nucleus segmentation package is available at: bitbucket.org/jstegmaier/xpiwit/downloads/. 3D contractility code is available on GitHub at: github.com/christophmark/jointforces.
