# Supplementary material for: Vimentin promotes collective cell migration through collagen networks via increased matrix remodeling and spheroid fluidity
Source: Commun Biol. 2026 Jun 18;9:1293. doi: 10.1038/s42003-026-10506-3 (PMC13639091; doi:10.1038/s42003-026-10506-3)
Supplement: Supplementary file 1 [file 42003_2026_10506_MOESM1_ESM.pdf]

Supplemental Information

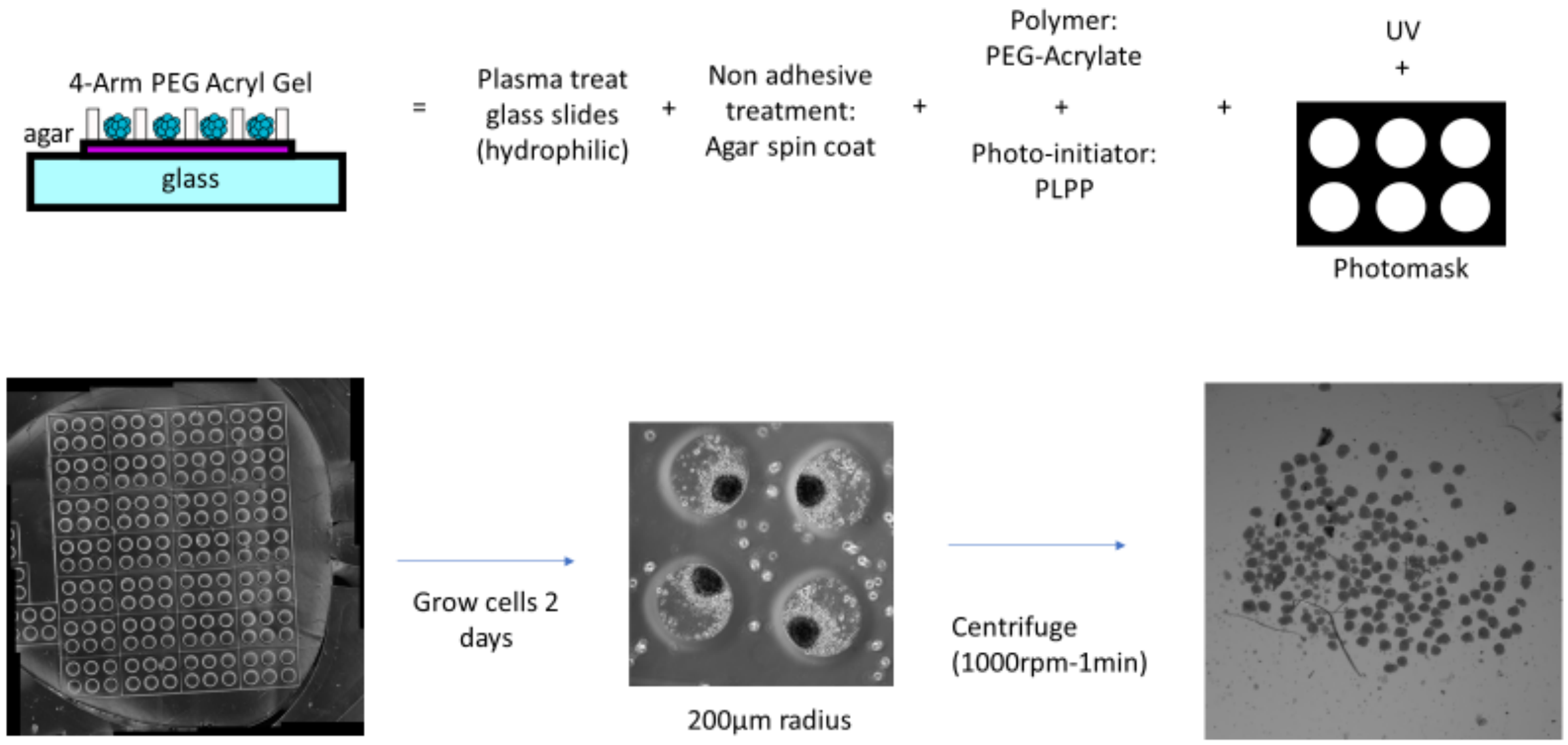

Figure S1. Schematic and workflow of creating microwell and spheroids

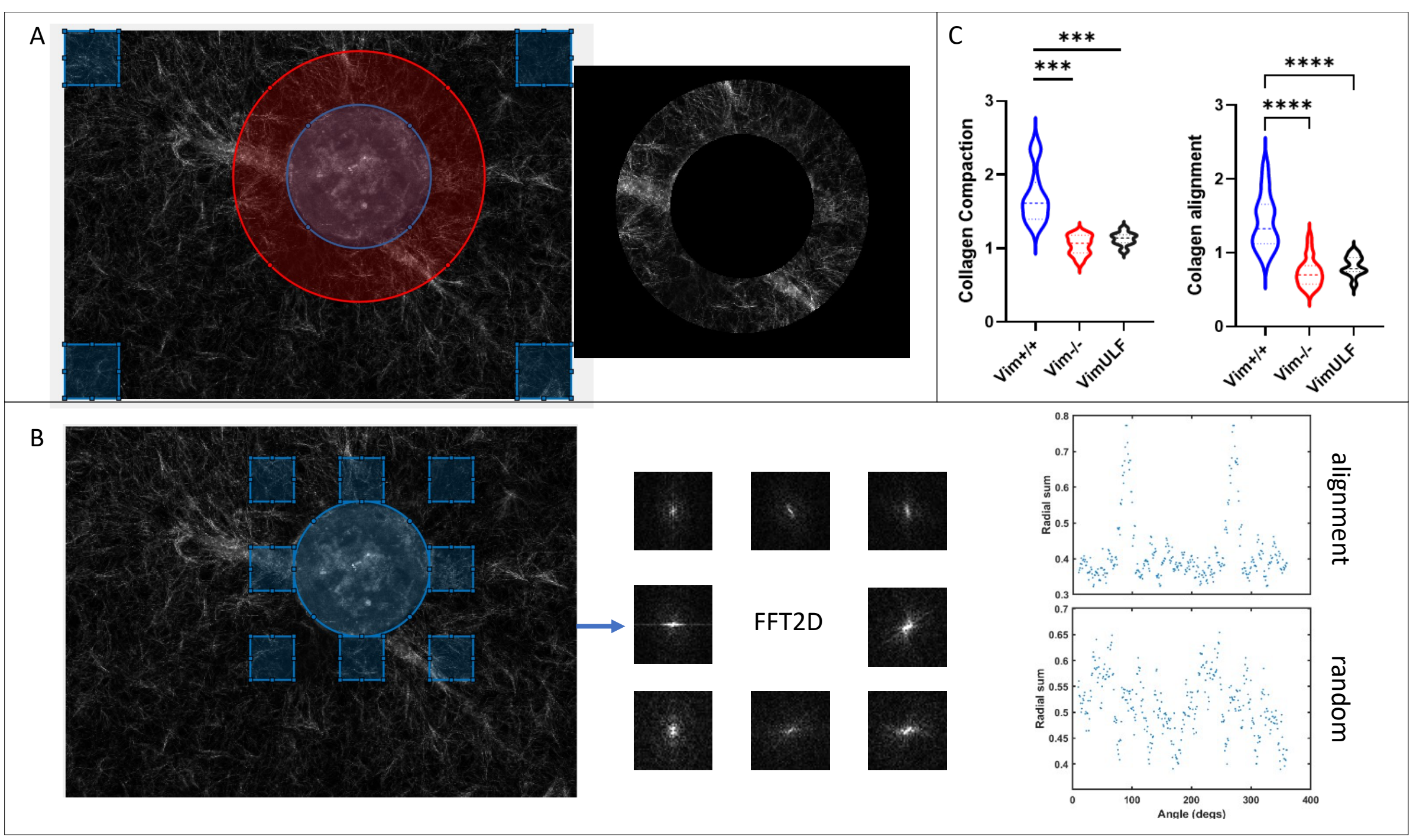

Figure S2. A) Characterization of collagen compaction. Collagen reflection intensity around the spheroid (100 um) was normalized and compared between different phenotypes. B) Collagen alignment analysis demonstration: area around the spheroid was extracted and Fourier transformed to check to alignment. Radial sweep of FFT area will contain high peaks used for calculation of alignment index. C) Graph of compaction and alignment including all phenotype Vim+/+, Vim-/-, VimULF (n = 3 independent experiments, N ≥ 24 spheroids for each condition)

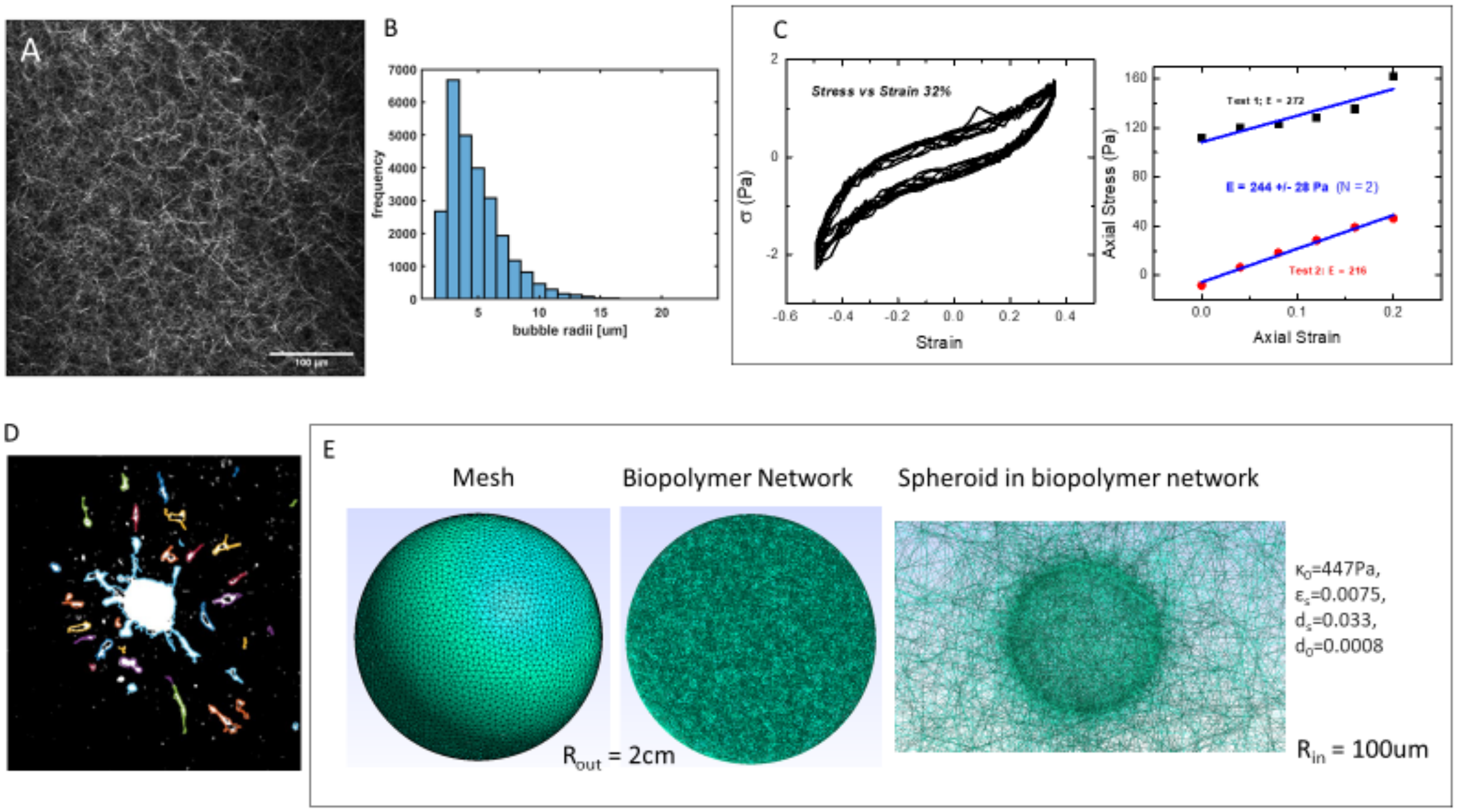

Figure S3. A) Confocal reflection image of collagen gel 1.5mg/ml. Scale bar = 100μm. B) Pore size distribution of the collagen gel. C) Frequency sweep of the collagen gel and Young's modulus fitting. D) Cross-sectional area analysis of the spheroid in MATLAB. Area of the spheroid only count the core and visibly attached protrusion. E) Collagen gel model for FEM constructed from saenopy package including parameters.

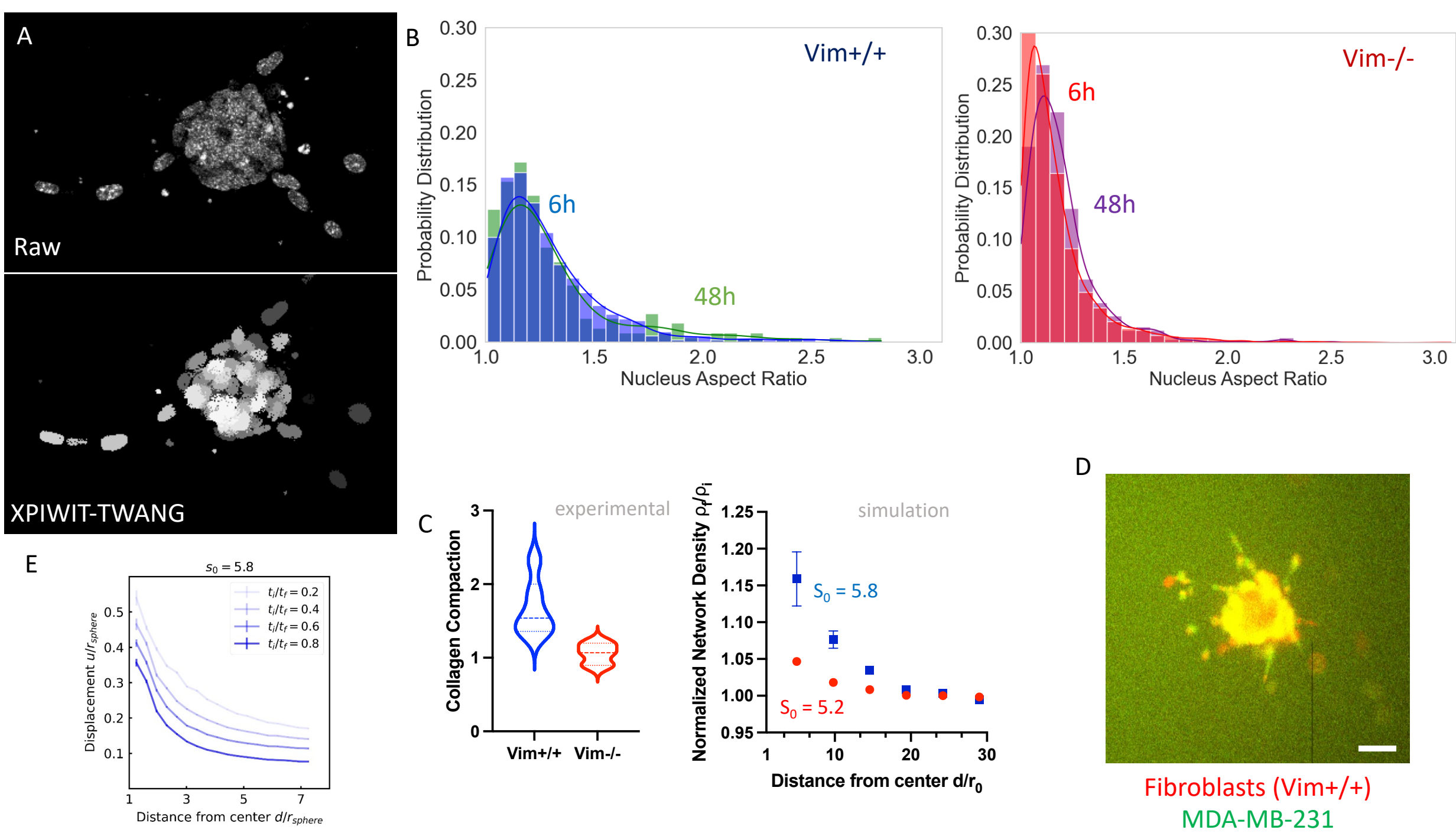

Figure S4. A) Z-projected nucleus-stained of raw and TWANG segmented images of a spheroid. B) Distribution of nuclei shape of Vim+/+ (Left) and Vim-/- (Right) at 6 and 48 hours. As time increases, Vim+/+ population becomes significantly more elongated compared to Vim-/-. C) Experimental collagen compaction (Left) and Simulated Network Density as a function of distance from the center (Right) (n = 3 independent experiments, N ≥ 8 spheroids for each condition). Error bars represent SEM. D) Membrane-live-stained of coculture MDA-MB-231 and mEFs spheroids (Green: MDA-MB-231, Red: mEFs). Scale bar = 50µm. E) Plot of normalized displacement u/r_{sphere} against normalized distance away from the center r/r_{sphere} for different choice of simulation time.

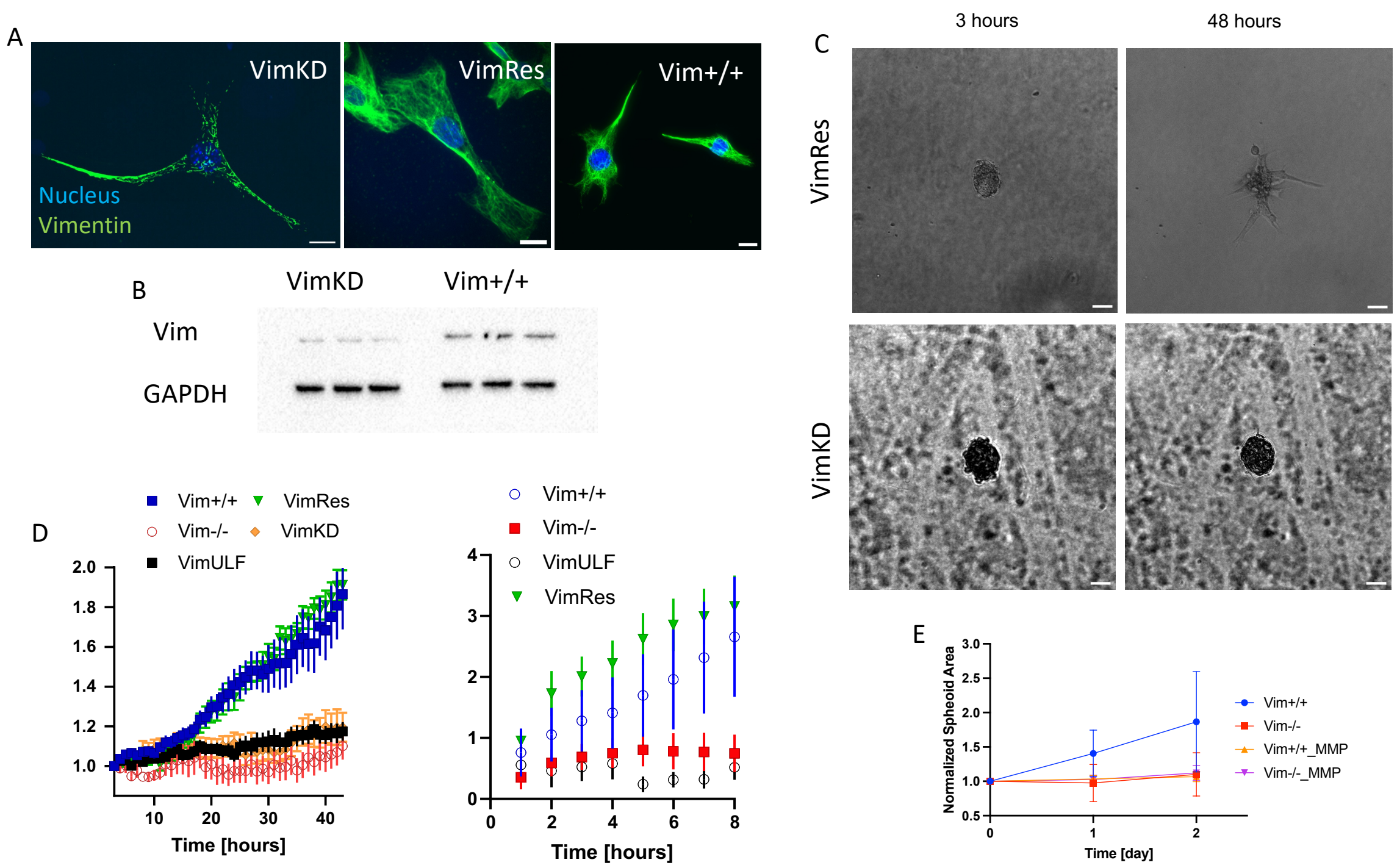

Figure S5. A) Fluorescent Stain of Vimentin knockdown wild-type cells (VimKD) and Vimentin Rescue in vimentin-null cells (VimRes) and wild-type cells (Vim+/+) showing broken VIF network in the VimKD cells while the VIF is intact in the VimRes cells similar to Vim+/+ cells (scale bar = 20 µm). B) Western blot of vimentin knockdown fibroblasts compared to wild-type cells. C) Morphology of VimKD and VimRes spheroids at 3 hours and 48 hours, showing VimKD spheroids has reduced invasiveness and VimRes recovers the cell invasion into collagen gel. Scale bar = 100µm. D) Left: Normalized spheroid area of Vim+/+, Vim-/-, VimULF, VimKD and VimRes (n ≥ 3 independent experiments, N ≥ 14 spheroids for each condition, Error bars represent SEM). Right: Contractility of Vim+/+, Vim-/-, VimULF and VimRes preinvasion (n = 3 independent experiments, N ≥ 8 spheroids for each condition, Error bars represent SD). E) Normalized spheroid area of collagen embedded wild-type, vimentin-null spheroids with and without MMP inhibitor in cell media (n ≥ 3 independent experiments, N ≥ 22 spheroids for each condition, Error bars represent SD). MMP inhibition antagonizes invasion of both spheroid population.

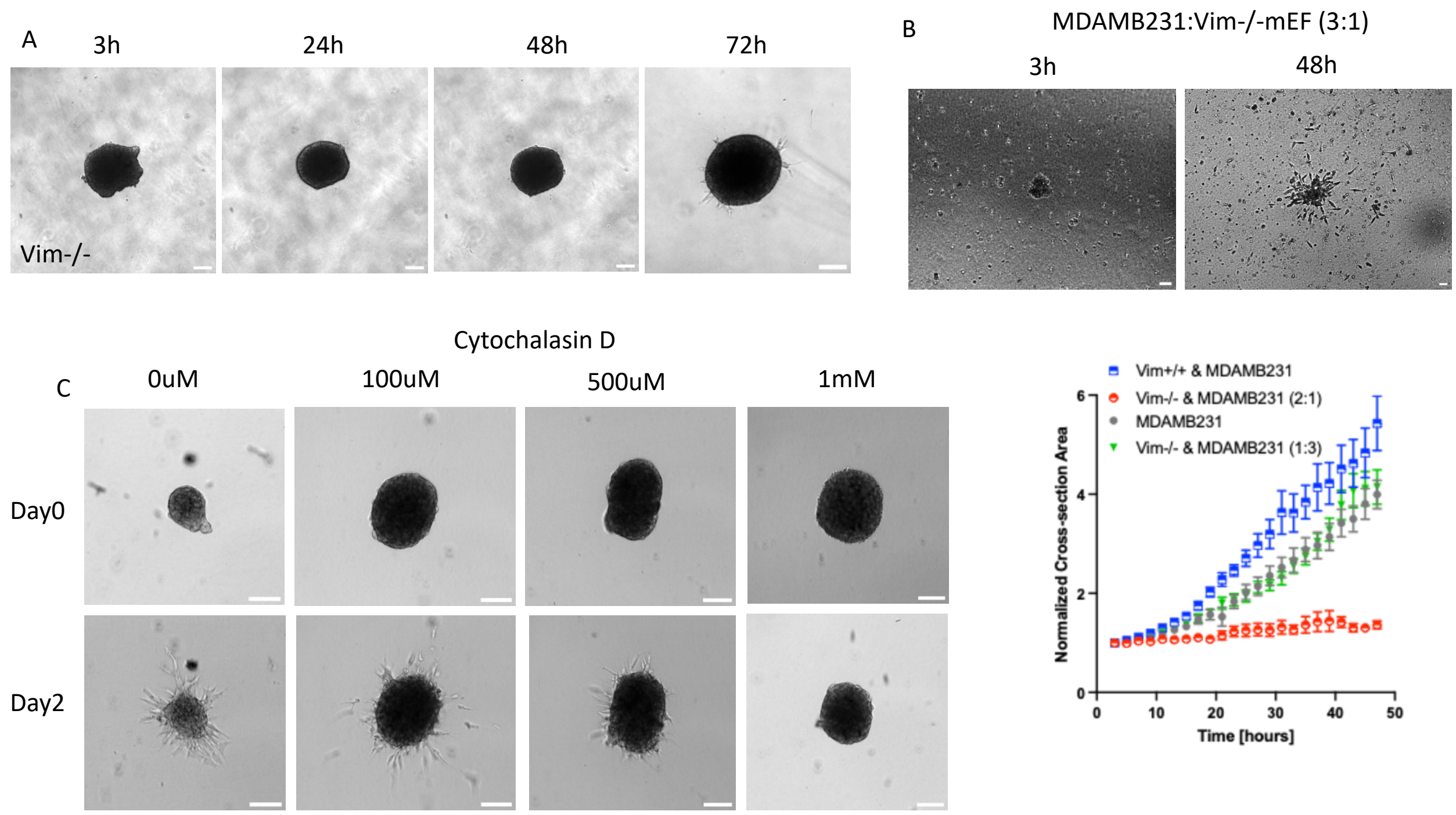

Figure S6. A) Image of a Vim-/- spheroid in collagen gel over 72 hours. Scale bar = 100μm. B) Images of a coculture Vim-/- and MDAMB231 at 1:3 ratio, graph of Vim+/+ mEFs and MDAMB231, MDAMB231, and Vim-/- mEFs and MDAMB231 at (2:1) and (1:3) ratio over 48 hours (n = 3 independent experiments, N ≥ 9 spheroids per condition, Error bars represent SEM). Scale bar = 50μm. Increasing the MDAMB231 cell number in the Vim-/- MDAMB231 coculture recovers the invasiveness of the spheroids. C) Images of Vim+/+ spheroid treated with Cytochalasin D at different doses over 2 days. Scale bar = 100μm

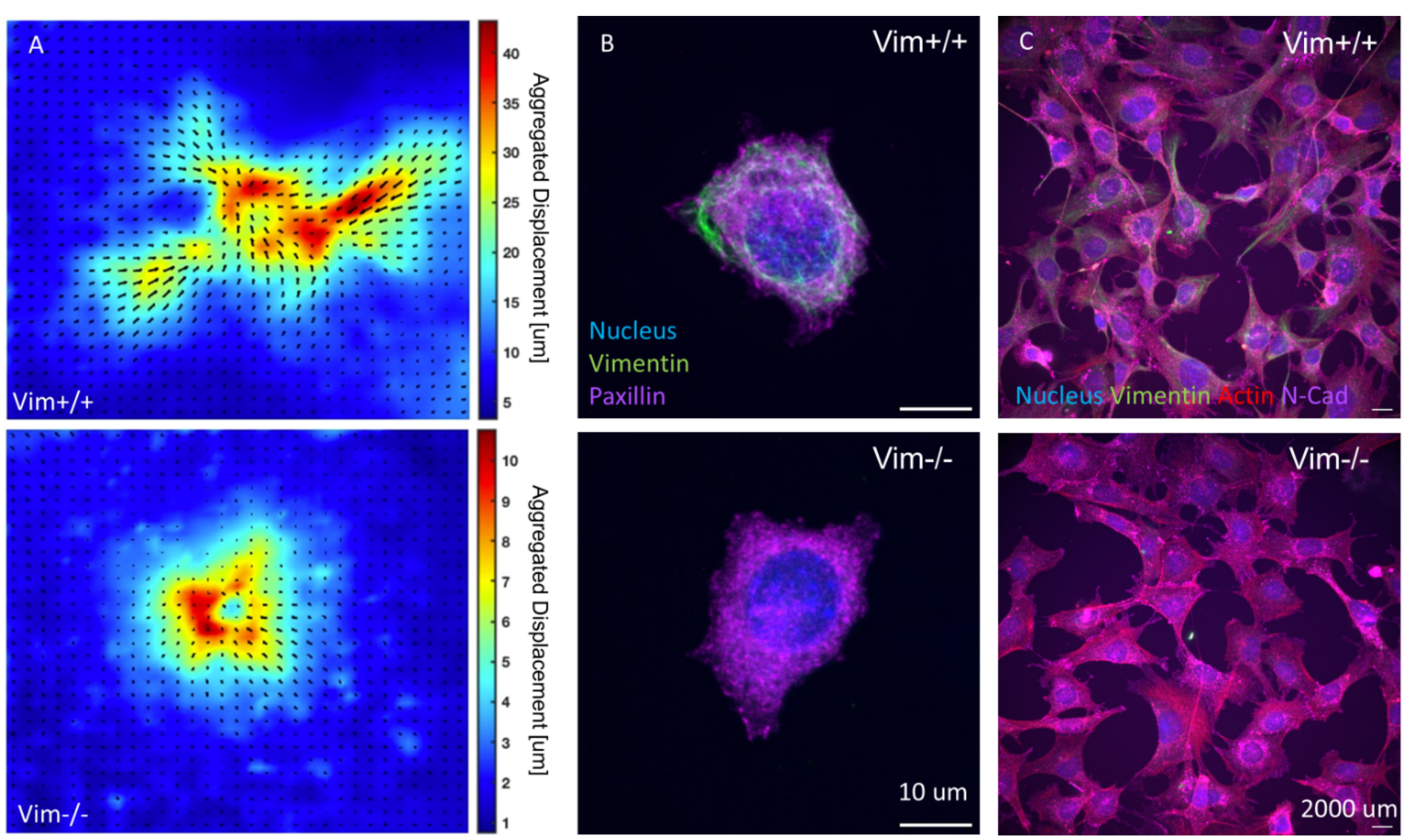

Figure S7. A) Aggregated displacement field and magnitude color map of a Vim+/+ and Vim-/- after 24 hours. B) Fluorescent stain of wild-type and vimentin-null cells on 500 Pa collagen coated polyacrylamide gels (Blue: Nucleus, Green: Vimentin, Magenta: Paxillin). There's no difference in cell-matrix adhesion (paxillin) signal. C) Fluorescent stain of wild-type and vimentin-null cells on collagen coated glass (Blue: Nucleus, Green: Vimentin, Magenta: N-Cadherin). There's no difference in the cell-cell adhesion (N-Cad) fluorescent signal.

Supplement Movie 1. Vim+/+ spheroid spreading in 3D collagen gel (1.5mg/ml) from 3-42 hours after seeding. (Scale bar = 100 μm)

Supplement Movie 2. Vim-/- spheroid spreading in 3D collagen gel (1.5mg/ml) from 3-42 hours after seeding. (Scale bar = 100 μm)

Supplement Movie 3. Vim+/+ spheroid beads displacement map in 3D collagen gel (1.5mg/ml) for 24 hours. (Scale bar = 100 μm)

Supplement Movie 4. Vim-/- spheroid beads displacement map in 3D collagen gel (1.5mg/ml) for 24 hours. (Scale bar = 100 μm)

Methods SI

Immunofluorescence Staining. Spheroids embedded in collagen were washed 3x in PBS supplemented with $CaCl_2$ and $MgCl_2$ and fixed with 4% paraformaldehyde (Sigma) for 30 min at room temperature, permeabilized with 0.5% Triton X-100 in PBS (PBS-T) at 4^0C for overnight. Next, spheroids were stained with primary antibody (rabbit anti-Vimentin (Abcam)) in blocking buffer at 4^0C overnight. After that, samples were washed 5 times (10 minutes each) and then incubated in optical clearing solution (80% Glycerol in PBS) at 4^0C overnight. Next, spheroids were stained with secondary antibody (anti-rabbit 488) in blocking buffer at 4^0C overnight. Blocking buffer includes 1% Tween-20, 1% Triton-X, 1% DMSO, 0.1% Sodium Azide and 1% BSA in PBS solution (adapted from Adhya et al. [1]). Samples were then again washed 5 times and incubated in optical clearing solution at 4^0C overnight. Next, spheroids were counterstained with Rhodamine Phalloidin (Alexa) at 1:300 dilution and DAPI 2 μg/ml in 40% DMSO:PBS solution at 4^0C overnight. Finally, samples were washed 5 times and incubated in optical clearing solution at 4^0C overnight before getting mounted for imaging. For 2D cell staining, cells were washed 3x with PBS and fixed with 4% paraformaldehyde for 15 mins before permeabilized with PBS-T for 30 minutes. Next cells were stained with primary antibody (rabbit anti-Vimentin (Abcam)/mouse anti-Paxillin (BD Biosciences)/mouse N-Cadherin antibody (3B9) (ThermoFisher)) in 1% BSA solution for 1 hour. Next, cells were then stained with corresponding secondary antibody (Alexa Fluor 488/555/568/647) for 1 hour in 1% BSA solution before counterstained with DAPI (1:2000 diluted in PBS) for 30 minutes.

Collagen Gel Pore Size Characterization. Collagen network was imaged with confocal reflection microscopy (CRM) on the LSM 980 (Zeiss) in reflected mode with 40x water immersion objective (LD LCI Plan-Apochromat 40x/1.2 Imm Corr DIC M27, Zeiss) (Fig S3A). The excitation laser wavelength is 488nm and backscatter was collected via beam splitter BC80/20. Confocal image stacks (50 μm) of four samples were captured at z-resolution of 0.25 μm. Pore size of the collagen gel (Fig S3B) was analyzed by a slight modification of the FIbeR

Extraction MATLAB code used in Stein et al. [2] to include bubble analysis algorithm by Munster & Fabry et al. [3]. In brief, the 3D image stack is first smoothed with a gaussian filter then binarized with appropriate threshold. Next, minimal distance from a fiber pixel to a background pixel is calculated using MATLAB's bwdist.m function in the image processing toolbox. The distance is then smoothed with a Gaussian filter. From this point, we feed the smoothed distance function to bubble analysis code and extract the pore size.

Collagen Gel Rheology. All rheology tests were performed with a Malvern Panalytical Kinexus Ultra+ rheometer (Malvern Panalytical) using a 20 mm parallel plate geometry. Collagen hydrogel solutions were mixed and immediately cast onto the bottom plate of the rheometer. The upper plate was then lowered to a gap height of 1mm. The gelation was tracked over the course of 30 min using a single frequency oscillatory sweep (f = 1Hz, ε = 2%) test at a temperature of 37°C. The gelation was monitored by the G' and G'', which reached a plateau value at around 20 min. For compression tests, samples were subjected to stepwise lowering of the top plates, corresponding to increasing steps of 4% compressive strain up to 20% strain. At each compressive strain %, the sample was allowed to equilibrate for 5min. All compression tests were performed with a continuous single frequency oscillatory sweep (f = 1Hz, ε = 2%). These compression tests were also used to estimate the Young's Modulus E of the sample. First, the final normal force value at each compressive step was used to calculate axial stress. Then plotting axial stress vs axial strain, a linear fit was applied where the slope provides an estimate of Young's Modulus E (Fig S3C).

Western Blot. Cells were plated in 6-well plates and lyses with HSEG buffer pH 7.4: 40 mM NaCl (Fischer Scientific), 5 mM EDTA (Fischer Scientific), 4% glycerol (Fischer Scientific), 20 mM NaF (Fischer Scientific), 1% Triton X-100, 1X protease inhibitor; 0.1 mM PMSF). Protein concentrations were determined using Bio-Rad Protein Assay Kit II (Bio-Rad Laboratories). Standard western blot procedure was performed. Nitrocellulose membranes were probed with primary rabbit anti-vimentin (Abcam) diluted in TBS-Tween20 (ThermoFischer) and incubated overnight at 4°C. The membranes were then incubated using rabbit HRP-conjugated secondary antibody (Boster Bio) for 1 h at room temperature. The protein levels were visualized using Clarity TM western ECL (Bio-rad Laboratories) substrate and imaged using Bio-Rad ChemiDoc TM imager.

Simulation Model. The spheroid is represented computationally as a 3D vertex model, described by the energy functional:

$$E_{VM} = K_V \sum_j (V_j - V_0)^2 + K_S \sum_j (S_j - S_0)^2 + \Gamma \sum_\alpha \delta_{\alpha,\beta} A_\alpha$$

where V_j and S_j represent the volume and surface area of the *j*-th cell, respectively. K_v and K_s are volume and area stiffness coefficients. Together with the target cell volume V_0 and target cell surface area S_0, these constants capture the cell's bulk elasticity and actomyosin cortex's isotropic contractility, respectively. Finally, the last term sums over the boundary polygons (with areas A_0) of the spheroid, with Γ representing the interfacial tension between the spheroid and the fiber network. In our simulations we use a ratio of stiffnesses KV /KS = 10. Specifically, we set K_V=10, K_S=1. We further set target volumes V_0=1 and interfacial tension coefficients Γ=1.

The collagen, constituting the extracellular matrix, is represented using a lattice model for a disordered elastic fiber network [4]. Specifically, the fiber network is initialized by randomly

linking nearest neighbor nodes on an FCC lattice with some finite probability p. Each node is then split into three sub-nodes (“phantom nodes”). The edges from each original node are then redistributed to the three sub-nodes, such that each sub-node connects to at most four edges (corresponding to two fibers, each contributing two segments). This construction effectively limits the local coordination number to ≤4.

The collagen, constituting the extracellular matrix, is represented using a lattice model for a disordered elastic fiber network. Specifically, the fiber network is initialized by randomly linking nearest neighbors on a FCC lattice with some finite probability. Each node is then split into three phantom nodes so that there is a maximum of four each per phantom node. Connected nearest-neighbor bonds that are initially parallel to the principal axes of the FFC lattice constitute fibers in our model. The energy functional of the fiber network is given by

$$E_{FB} = \frac{k_S}{2}\sum_{\langle ij\rangle} f_{ij}(l_{ij} - l_0)^2 + \frac{K_B}{2}\sum_{m=1}^{3}\sum_{\langle ijk\rangle=\pi_0} f_{ij}f_{jk}(\theta_{ijk}^{m} - \pi)^2$$

where, $f_{ij} = 1$ if a bond is occupied and $f_{ij} = 0$ if not. The length of the bond <*ij*> is l_{ij}. These bonds have length stiffness KS and natural length l0. Furthermore, θ_ijk^m represents the angle between nearest neighbor bonds that are crosslinked by the same phantom node with phantom node index m. These angles have bending stiffness KB.

In our simulations, the fiber networks are generated from the FCC lattice with a nearest neighbor occupation probability p = 0.85. We use a ratio of stiffnesses KS /KB = 10000. Specifically, we set K_S=10, K_B=0.001. Furthermore, in terms of the length scale set by the sides of the FCC lattice unit cell, we set natural lengths of the fiber edges l_0=2.8284271.

A fixed number of surface polygons on surface cells of the spheroid are connected to nearby fiber ends on the network via active linker springs. These linker springs model focal adhesions between the cells and the collagen matrix. At the beginning of the simulation, the natural lengths of these linker springs shrink at a constant rate to mimic the molecular clutch mechanism for focal adhesions.

Cell rearrangements are a central component of the model because they allow a confluent tissue to flow and reorganize while maintaining its integrity. In two dimensions, these neighbor-exchange motions are known as T1 transitions, and in three dimensions they take the form of reconnection events. Prior work has established algorithms for such 3D reconnections, focusing on when short edges transition into triangular faces and vice versa once their maximum edge length falls below a threshold. To ensure that these events represent physically valid cell rearrangements, additional constraints are imposed: the moves must be geometrically, energetically, and topologically reversible. Recent refinements include preventing two polyhedral cells from simultaneously sharing multiple faces, which would violate topological reversibility.

Beyond changes in cell connectivity, the model evolves through overdamped Brownian dynamics applied to each cell vertex. The motion of any vertex is described by a balance of conservative forces - arising from area and volume constraints, cell–cell interactions, interfacial tension between the spheroid and surrounding fiber network, and active linker springs - and stochastic forces representing internal cellular fluctuations. These fluctuations are encoded

through an effective temperature, which is small compared with the deterministic force contributions but helps the system explore the underlying energy landscape. Vertex positions are updated using the Euler–Maruyama scheme, while the fiber-network nodes evolve via overdamped Euler integration with no active noise term.

Finally, to construct the initial geometry, a bulk Voronoi tessellation is generated, from which a spherical cluster of cells (a “spheroid”) is extracted. A surrounding fiber network is carved out to accommodate the spheroid, and active linker springs are assigned along a chosen fraction of boundary faces. After a specified relaxation period, these springs begin contracting toward their reduced target length, driving the coupled evolution of the tissue and fiber network. Simulation parameters are selected based on earlier fiber-network studies and computational efficiency, with conversions to biophysical scales provided (e.g., one simulation length unit ≈ 10 μm, one simulation time unit ≈ 0.144 s). For clarity, all results are reported in dimensionless form.

Simulation Data: The simulation starts with randomly generated configurations for the spheroid and fiber network, an appropriate delayed initial time point is chosen for taking measurements. For all samples, the average displacement and resulting densification of fiber nodes are measured with respect to the states from this time, up to the final states of the samples. Aside from avoiding the initial random configurations, this initial time point is also chosen to be after the active contraction of the linker springs is complete, with these active pulling forces having caused rearrangements in the spheroid and fiber network. Further dynamics of the system past this initial time point is due overdamped motion, with added random activity in the spheroid. A plot of displacement graphs with different choices of time points in Fig S4E.

1. Adhya, D., et al., *Application of Airy beam light sheet microscopy to examine early neurodevelopmental structures in 3D hiPSC-derived human cortical spheroids.* Mol Autism, 2021. **12**(1): p. 4.
2. Stein, A.M., et al., *An algorithm for extracting the network geometry of three-dimensional collagen gels.* J Microsc, 2008. **232**(3): p. 463-75.
3. Münster, S. and B. Fabry, *A simplified implementation of the bubble analysis of biopolymer network pores.* Biophys J, 2013. **104**(12): p. 2774-5.
4. Broedersz, C.P., M. Sheinman, and F.C. MacKintosh, *Filament-Length-Controlled Elasticity in 3D Fiber Networks.* Physical Review Letters, 2012. **108**(7): p. 078102.
