# Supplementary material for: Vimentin promotes collective cell migration through collagen networks via increased matrix remodeling and spheroid fluidity
Source: Commun Biol. 2026 Jun 18;9:1293. doi: 10.1038/s42003-026-10506-3 (PMC13639091; doi:10.1038/s42003-026-10506-3)
Supplement: Supplementary file 2 [file 42003_2026_10506_MOESM2_ESM.pdf]

Description of Additional Supplementary files

File name: Supplementary Data

Description: Source data behind the graphs in the paper

File name: Supplementary Movie 1

Description: Vim+/+ spheroid spreading in 3D collagen gel (1.5mg/ml) from 3-42 hours after seeding. (Scale bar = 100 μm)

File name: Supplementary Movie 2

Description: Vim-/- spheroid spreading in 3D collagen gel (1.5mg/ml) from 3-42 hours after seeding. (Scale bar = 100 μm)

File name: Supplementary Movie 3

Description: Vim+/+ spheroid beads displacement map in 3D collagen gel (1.5mg/ml) for 24 hours. (Scale bar = 100 μm)

File name: Supplementary Movie 4

Description: Vim-/- spheroid beads displacement map in 3D collagen gel (1.5mg/ml) for 24 hours. (Scale bar = 100 μm)
