# Supplementary material for: Vimentin promotes collective cell migration through collagen networks via increased matrix remodeling and spheroid fluidity
Source: Commun Biol. 2026 Jun 18;9:1293. doi: 10.1038/s42003-026-10506-3 (PMC13639091; doi:10.1038/s42003-026-10506-3)
Supplement: Supplementary file 3 [file 42003_2026_10506_MOESM8_ESM.pdf]

nature portfolio

Corresponding author(s): Minh Tri Ho Thanh

Last updated by author(s): May 20, 2026

Reporting Summary

Nature Portfolio wishes to improve the reproducibility of the work that we publish. This form provides structure for consistency and transparency in reporting. For further information on Nature Portfolio policies, see our Editorial Policies and the Editorial Policy Checklist.

Statistics

For all statistical analyses, confirm that the following items are present in the figure legend, table legend, main text, or Methods section.

n/a	Confirmed	
☐	☒	The exact sample size (*n*) for each experimental group/condition, given as a discrete number and unit of measurement
☐	☒	A statement on whether measurements were taken from distinct samples or whether the same sample was measured repeatedly
☐	☒	The statistical test(s) used AND whether they are one- or two-sided<br>*Only common tests should be described solely by name; describe more complex techniques in the Methods section.*
☒	☐	A description of all covariates tested
☒	☐	A description of any assumptions or corrections, such as tests of normality and adjustment for multiple comparisons
☐	☒	A full description of the statistical parameters including central tendency (e.g. means) or other basic estimates (e.g. regression coefficient) AND variation (e.g. standard deviation) or associated estimates of uncertainty (e.g. confidence intervals)
☐	☒	For null hypothesis testing, the test statistic (e.g. *F*, *t*, *r*) with confidence intervals, effect sizes, degrees of freedom and *P* value noted<br>*Give P values as exact values whenever suitable.*
☒	☐	For Bayesian analysis, information on the choice of priors and Markov chain Monte Carlo settings
☒	☐	For hierarchical and complex designs, identification of the appropriate level for tests and full reporting of outcomes
☒	☐	Estimates of effect sizes (e.g. Cohen's *d*, Pearson's *r*), indicating how they were calculated

Our web collection on statistics for biologists contains articles on many of the points above.

Software and code

Policy information about availability of computer code

Data collection	Images were obtained using Nikon NIS Element 5.10 and Zeiss Zen 3.3
Data analysis	Traction force was calculated using the jointforces package provided by Christoph Mark et al. at https://github.com/christophmark/jointforces.<br>Nucleus segmentation was done using software package XPIWIT available at https://bitbucket.org/jstegmaier/xpiwit/downloads/<br>Simulation of spheroid in collagen gel was done using model by Tao Zhang et al. available at https://doi.org/10.1101/2024.03.25.586590/<br>Images are analyzed using MATLAB 2024b<br>Statistic was performed using Prism v9

For manuscripts utilizing custom algorithms or software that are central to the research but not yet described in published literature, software must be made available to editors and reviewers. We strongly encourage code deposition in a community repository (e.g. GitHub). See the Nature Portfolio guidelines for submitting code & software for further information.

Data

Policy information about availability of data

All manuscripts must include a data availability statement. This statement should provide the following information, where applicable:

- Accession codes, unique identifiers, or web links for publicly available datasets
- A description of any restrictions on data availability
- For clinical datasets or third party data, please ensure that the statement adheres to our policy

Traction force software is available at: https://github.com/christophmark/jointforces. Nucleus segmentation software is available at: https://bitbucket.org/jstegmaier/xpiwit/downloads/. Model simulation is available at: https://doi.org/10.1101/2024.03.25.586590/. All the raw data in this study are available from the corresponding author on request.

Research involving human participants, their data, or biological material

Policy information about studies with human participants or human data. See also policy information about sex, gender (identity/presentation), and sexual orientation and race, ethnicity and racism.

Reporting on sex and gender	N/A
Reporting on race, ethnicity, or other socially relevant groupings	N/A
Population characteristics	N/A
Recruitment	N/A
Ethics oversight	N/A

Note that full information on the approval of the study protocol must also be provided in the manuscript.

Field-specific reporting

Please select the one below that is the best fit for your research. If you are not sure, read the appropriate sections before making your selection.

☒ Life sciences ☐ Behavioural & social sciences ☐ Ecological, evolutionary & environmental sciences

For a reference copy of the document with all sections, see nature.com/documents/nr-reporting-summary-flat.pdf

Life sciences study design

All studies must disclose on these points even when the disclosure is negative.

Sample size	Sample size is denotes as N in the manuscript and represents the total number of spheroids/cells for each group for each experiment.
Data exclusions	None
Replication	All experiments are repeated and the number of repetition is denoted as n in the manuscript. Repetitions are done on different date.
Randomization	N/A
Blinding	N/A

Reporting for specific materials, systems and methods

We require information from authors about some types of materials, experimental systems and methods used in many studies. Here, indicate whether each material, system or method listed is relevant to your study. If you are not sure if a list item applies to your research, read the appropriate section before selecting a response.

Materials & experimental systems

n/a	Involved in the study	
☐	☒	Antibodies
☐	☒	Eukaryotic cell lines
☒	☐	Palaeontology and archaeology
☒	☐	Animals and other organisms
☒	☐	Clinical data
☒	☐	Dual use research of concern
☒	☐	Plants

Methods

n/a	Involved in the study	
☒	☐	ChIP-seq
☒	☐	Flow cytometry
☒	☐	MRI-based neuroimaging

Antibodies

Antibodies used

Alexa Rhodamine Phalloidin (Invitrogen),
DAPI (Invitrogen),
rabbit anti Vimentin (Abcam),
mouse anti-Paxillin (BD Biosciences),
mouse N-Cadherin antibody (3B9) (ThermoFisher),
Alexa Fluor 488/555/568/647 (Invitrogen),
rabbit HRP-conjugated secondary antibody (Boster Bio)

Validation

Validation Statements provided by manufacturer.

Eukaryotic cell lines

Policy information about cell lines and Sex and Gender in Research

Cell line source(s)

Vimentin wild-type and vimentin knockout mouse embryonic fibroblasts (MEFs) were gifted by J. Eriksson from Abo Akademi.
Vimentin unit length filament cells were gifted by the Vladimir Gelfand lab from Northwestern University.
Vimentin Rescue mEFs were gifted by the Robert Goldman lab from Northwestern University.
MDAMB231 human breast cancer cells were given by the Mingming Wu lab from Cornell University.

Authentication

None of the cell lines was authenticated

Mycoplasma contamination

Cell lines have been tested for mycoplasma contamination.

Commonly misidentified lines
(See ICLAC register)

None

Plants

Seed stocks

N/A

Novel plant genotypes

N/A

Authentication

N/A
