# Supplementary material for: Vimentin promotes collective cell migration through collagen networks via increased matrix remodeling and spheroid fluidity
Source: Commun Biol. 2026 Jun 18;9:1293. doi: 10.1038/s42003-026-10506-3 (PMC13639091; doi:10.1038/s42003-026-10506-3)
Supplement: Supplementary file 4 [file 42003_2026_10506_MOESM9_ESM.pdf]

nature portfolio

Peer Review File

Vimentin promotes collective cell migration through collagen networks via increased matrix remodeling and spheroid fluidity

Corresponding Author: Dr Minh Tri Ho Thanh

This file contains all reviewer reports in order by version, followed by all author rebuttals in order by version.

Version 0:

Reviewer comments:

Reviewer #1

(Remarks to the Author)
This manuscript describes migration of mouse fibroblasts from a spheroid into a collagen gel. The authors were interested primarily in the effects of the intermediate filament vimentin, and they compared results for wildtype fibroblasts (which expressed vimentin) and fibroblasts from vimentin knockout mice. The main observations were that cells expressing vimentin showed more migration/invasion into the collagen gel and these cells reorganized and deformed the matrix more than vimentin null cells. The authors attributed these differences to a combination of MMP activity and fluidity within the spheroid, which was assessed through a computational 3D vertex model. The findings on effects of vimentin will likely be of interest to the field, but there are many open questions that the authors must address about this manuscript. The questions are listed below, approximately in order of importance.

1. Details of the 3D vertex model are not given in the manuscript. The manuscript cites a prior study, but the essential components of the model need to be included in the present manuscript. The results section should briefly describe the key components of the model. For example, are the cells given elasticity associated with volume and surface area as is common in a 3D vertex model? Are the fibers given elasticity in response to both stretching and bending, as is common in models simulating fibrous materials? How are the active cell forces simulated? How are the cells connected to the fibers? (Lines 303-304 make a specific claim about cells pulling on fibers, so the connection between cells and fibers seems to be important.) Full details of the model should be included in the methods section or supplemental information. These details are essential to understand the relevance and findings of the computational model, and, since they are not included in the first version of the manuscript, it is possible that new questions about the model will arise in a future round of review.

2. Lines 314-315 suggest that a more fluid-like spheroid causes more deformation and rearrangement on the surrounding collagen. How, specifically, does this occur in the computational model? The results in the present manuscript do not convey how this might be possible. Is there experimental evidence to support how the computational model treats the interaction between cells and fibers?

3. In Fig. 6d, the results from experiments and computational model are quite different. Specifically, the blue curve is slightly above the red curve for experiments, but for model, the blue is very substantially above red, especially for moderate and large distances from the center, which is notably different from the experimental data. Can model parameters be further tuned for better match? If no, is the model a relevant one for comparing to these experiments?

4. Additional clarity in writing is needed when describing matrix degradation. The last sentence of the abstract refers to “MMP transportation,” but the authors didn’t measure MMP production or transport and didn’t show those were affected by vimentin; instead, they showed that MMPs were important for invasion. In the first sentence of conclusion, the authors state, “our experimental results motivate a physical mechanism in which vimentin-mediated collagen degradation…,” but the data didn’t show that the degradation was mediated or affected by vimentin; instead, the data showed more invasion for vimentin-expressing cells and that degradation depended on MMPs. One possible explanation for these observations is that vimentin enhances degradation, but this was not shown by the data—in fact, the main support for the effects of vimentin on MMP activity comes from citing prior literature, and not the authors’ experimental measurements. The authors should revise the text to clarify what can be concluded based on their own experiments and what can be inferred by comparing to prior

literature. The authors are encouraged to read the other sections of the manuscript (such as results and paragraph 4 of discussion) to ensure accuracy of description of the results from this manuscript and a clear distinction made between conclusions that can be drawn directly from this manuscript and conclusions made by integrating results from this study and others.

5. The authors use the words “traction,” “traction force microscopy,” and “contractility” (with units of force), but the methods used are questionable for quantifying cell tractions or forces. The constitutive relationship used for the collagen gel is not stated, and it's still an open question in the field as to what constitutive relationship to use for fibrous materials. The data reporting contractility also used 2D imaging, despite this being a 3D system, which creates problems in computing the tractions (and, in turn, the contractility shown in Fig. 3c). The approach to quantify contractility assumes a sphere, but for wildtype cells, there are invading strands, meaning the system is not spherically symmetric. For the purposes of this study, showing bead displacements is sufficient. The authors should keep the displacement data but remove the contractility data and remove the word traction from the manuscript.

6. Fig. 5d shows interesting results of cells migrating into pre-made tracks, but the images are low contrast and it is difficult to see the cells. Can the authors provide higher contrast images? Additionally, can the authors quantify the results, e.g., distance of migration or something similar?

7. In Fig. 6d, it is unclear how the displacement data is normalized. The figure axes say u/r_0 (presumably r_0 is the initial spheroid radius) but values are greater than 1. If the displacement is inward, a value of 1 would mean that the displacement is equal to the spheroid radius, i.e., the spheroid has collapsed to a point. Values greater than 1 shouldn't be possible. Can the authors check the normalization and define it in the caption?

8. In Fig. 7, it looks like the initial size of the spheroid is different in the sample images in 7b. I see 7c normalizes by initial area, but if the initial size is drastically different, as it is here, there might be fewer cells that can invade, which would in turn limit the total spheroid area. Are the initial sizes substantially different?

9. On line 145, the authors state that the maps of displacement field correlate with regions of invading strands of cells. The image shown in Fig. 3 shows only the displacement maps. Not the cells. The authors should add images of the cells for this experiment so that the data support the correlation between cell invasion and locations of displacements.

10. Line 176-177 says “Our results thus far demonstrate that vimentin plays an important role in collective migration of fibroblast cells through collagen networks.” This wording is not quite accurate. The data show that cells with vimentin migrate more than cells without vimentin, i.e., migration correlates with vimentin. But the wording implies that vimentin is the cause. The authors should revise this wording to be more precise in describing the results.

11. Line 291 refers to a “time point sufficiently large enough to allow for both the cells and fibers to deform….” Can the authors provide more detail on how the time point was determined? The following text refers to a “new equilibrium”—does this text about time have something to do with the computational solver or is it a meaningful representation of time in the experiments? The text here also refers to supplemental information, but the supplemental information does not appear to have any information about the time point selected to show.

12. Starting in paragraph 2 of the discussion, the authors talk about how the findings are surprising. One finding not included in this section is that vimentin correlates with greater cell aspect ratio. Isn’t this finding an expected one, given that vimentin is often correlated with mesenchymal phenotypes?

13. The authors use the word “polar” or “polarized” in the abstract, results, and discussion. The word polar implies a vector quantity with a front and back. The experiments and computational model do not quantify a front or back of the cell; instead they quantify an aspect ratio. Instead of using the word “polar,” wording that conveys aspect ratio would be more accurate.

14. The last sentence of the abstract mentions that vimentin has an effect of “enhancing migratory persistence,” but the authors didn’t quantify migratory persistence in this manuscript. They quantified the total amount of invasion, but not how persistent the migration was. The wording should be updated for more accuracy.

15. In the images of fibers measured by confocal reflectance in Fig. 2, the contrast is low, and it is difficult to see alignment and density of the fibers. Can the authors provide higher contrast images in the main text?

16. Text near line 295 refers to collagen strain, but the figures show only displacement. Text about strain should be removed.

17. In Fig. 1c, the resolution of the images is low. It is difficult to see the localization of actin and vimentin in the cells. Can the authors provide higher resolution images?

18. The caption to Fig 6c says that the results show nucleus aspect ratio, but the results from the model show cell aspect ratio, not nucleus aspect ratio.

19. Fig 2b has two * symbols; it appears only one is needed for a statistical comparison. Is one an error?

20. Line 251 has a typo: “spheroid” should be “spheroids”

21. Figure 8 does not appear to be mentioned in the main text.

Reviewer #2

(Remarks to the Author)
In this manuscript, Minh Tri Ho Thanh and colleagues investigate the role of vimentin in fibroblast invasion using in vitro 3D collagen models. They compare wild-type and vimentin-null mouse embryonic fibroblasts (mEFs) to assess their capacity for collagen matrix invasion, collagen remodeling, traction force generation, and spheroid fluidity. Their results indicate that loss of vimentin impairs MMP-dependent invasion, decreases collagen remodeling and traction forces, and alters the mechanical properties of spheroids. Interestingly, they also suggest that vimentin-deficient fibroblasts are less effective in promoting adenocarcinoma cell invasion.
While the role of vimentin in promoting cell migration and invasion is well established, the manuscript presents some novel and potentially impactful findings—particularly those related to spheroid fluidization and the cooperative invasion of cancer cells. However, most of the results are correlative and mechanistic aspects remain unclear. The study also lacks essential experimental controls, such as rescue experiments and siRNA-mediated knockdowns, which limits the strength of the conclusions.

Major Concerns
1- Lack of validation via complementary approaches
The study relies exclusively on vimentin-null mEFs. The authors should confirm their findings using siRNA-mediated knockdown in wild-type cells and rescue experiments. While Figure 4 presents data using a mutant vimentin unable to form filaments (ULF), it remains critical to demonstrate that re-expression of wild-type vimentin restores invasion capacity, contractility, fluidization.

2- Weak mechanistic insight
The authors propose that impaired invasion results from increased contractility leading to decreased fluidity and altered matrix remodeling. However, these hypotheses remain untested. Multiple alternative explanations should be considered and experimentally addressed:
- Does vimentin expression affect cell proliferation or survival within spheroids?
- Are cell adhesion or integrin expression altered in vimentin-null cells?
- Given that invasion appears MMP-dependent and vimentin has been linked to MT1-MMP secretion, is MT1-MMP expression or localization altered?
- Is the reduction in spheroid fluidity (cell/nuclear shape) a cause or a consequence of impaired invasion?
- Are decreased contractility and matrix alignment causes or a consequences of impaired invasion? Does mild inhibition of contractility reduce invasion in wild-type cells?

Specific concerns
1. Figure 1. Are vimentin-null cells entirely incapable of invading, or is their invasion only delayed? Long-term assays (e.g., >72h) would clarify this.
2. Figure 2. Can the authors provide representative images of vimentin-null spheroids with protrusions, as seen in ~50% of cases (Figure 5b)? Do these spheroids show any degree of collagen remodeling?
3. The recent study (PMID: 39952241) reporting vimentin-dependent regulation of MT1-MMP in macrophages should be cited and discussed. Do mEFs form podosomes, and are these structures affected by vimentin loss?
4. Figure 3a. The spheroid boundary and scale bar should be marked. In the WT condition, it is difficult to identify the spheroid center.
5. Figure 3b. The x-axis scale is unclear—possibly logarithmic? A clear label and indication of the zero point are needed.
6. Figure 4.
a. The analysis at 24h should be extended to 48h for consistency with other figures.
b. Presenting the data as histograms is also inconsistent with the curves used in Figures 1 and 2. Time-course curves would be more informative.
c. This figure should include data from vimentin siRNA/shRNA knockdowns and wild-type rescue experiments.
d. Figure 4d includes statistical analysis with only two independent replicates, which is insufficient.
7. Figure 5. The authors should normalize the normalized spheroid spreading area as in the other figures.
8. Figure 5c, 5d. Laser-Ablated Channel Assay. The qualitative result is compelling but lacks quantification. What proportion of total invading cells enter the channel in each condition? What is the absolute number of cells in channels per condition?
9. Figure 6. Conclusions about spheroid fluidity are based on nuclear deformation, whereas fluidity should relate to cell shape. The comparison between experimental nuclear aspect ratios and simulated cell shapes is not well justified. Notably, nuclei (shown in a) appear elongated along the spheroid surface, rather than perpendicular to it, which, to me, contradicts the proposed mechanism where fluidization facilitates invasion.
10. Figure 7. The description of observations is vague and lacks quantification. Specific questions need to be addressed:
a. Do mEF and MDA-MB cells proliferate at the same rate? Is the 1:2 ratio maintained over time?
b. Are both cell types homogeneously mixed, or is there evidence of cell-type-specific segregation?
c. Which cells are invading—mEFs, MDA-MBs, or both? Are WT fibroblasts leading cancer cells during invasion?
d. Could reduced invasion in mixed spheroids with vimentin-null fibroblasts simply result from the large presence of a non-invasive (vimentin null mEF) population?

Version 1:

Reviewer comments:

Reviewer #1

(Remarks to the Author)
The revised manuscript and point-by-point response have addressed most of the comments from the first review, though some open questions still remain. Numbers below correspond to the same numbers in the first review.

1. Question 1 of the first review was to give details about the model. Those details were added to the supplemental information, but some open questions still remain that are important to describe the model effectively:
- It is not clear what is meant by the sentence “Each node is then split into three phantom nodes so that there is a maximum of four each per phantom node.” The meaning of a phantom node is not clear, and it is not clear what “four each” is referring to. Is this referring to how fibers connect to nodes? This should be revised to give specific details, because it is well established that the number of fibers that connect at each node (often referred to as the “coordination number”) is a very important factor in the behavior of matrix materials.
- Equations are given for the simulations, but the simulation parameters are not stated. Specific parameters are K_B, k_s, K_v, V_0, K_s, S_0, and Lambda. The text says the parameters are based on prior studies, but no citations are given. It is necessary to state the simulation parameters and demonstrate (with citations) that they're within the range of prior studies.

3. Question 3 was about comparison between experiments and modeling of matrix displacements over distance from the center of the spheroid. The updated Fig 6 still does not show model results that convincingly capture the experiments. In the updated figure 6D, the red line for the model is still notably different than in the experiments (in the model, it's still notably below the blue curve). The authors suggest that a different time step and/or other values of S_0 can improve the comparison--this should be done with updated model results presented in main text OR the main text should acknowledge that there is quantitative disagreement between experiments and model and also describe why/how the qualitative trends in from the model are sufficient to interpret the experiments.

5. Question 5 was about how tractions were calculated from the experiments. It is good that the language has been changed from “traction” to “contraction” and it is good that details have been added about the methods. Two remaining points are:
- The description of the method talks about "fibers" but the approach is a continuum approach, meaning it doesn't actually account for single fibers. (The term "semi-affine" has been used by some prior manuscripts and gives the impression that this approach accounts for individual fibers, but it does not; the method originally developed by Steinwachs et al. uses a continuum approach.) Given that spheroids are much larger than the fiber lengths, a continuum approach is probably fine, but the authors should revise the text in the methods section describing the method to clarify that this method treats the matrix as a continuum.
- The model has various constants. According to the documentation on Github, there are 4:
K_0: the linear stiffness (in Pa)
D_0: the rate of stiffness variation during fiber buckling
L_S: the onset strain for strain stiffening
D_S: the rate of stiffness variation during strain stiffening.
The constants used need to be stated.

Reviewer #2

(Remarks to the Author)
The authors have satisfactorily and substantively addressed nearly all rmy previous concerns. The revision presents a coherent, well supported story linking vimentin to collective migration through MMP dependent remodeling, and spheroid fluidization. I just have two minor points which I think should be addressed before publication.
1- In the rescue experiments, re-expressed vimentin in vimentin-depleted mEFs appears as a fragmented/patchy network rather than a typical extended filament meshwork. Yet, those cells recover the “less-fluidized” phenotype and regain aspects of the vimentin-dependent migratory program. It would strengthen the manuscript to explicitly discuss how a fragmented vimentin network could mechanistically restore function. Can it be a mechanical function or does it have a signaling role. If feasible within scope, a brief quantification (added to Methods/Supplement) would help: e.g., filament length in the rescue lines, and whether these correlate with rescued contractility/MMP activity. In absence of new experiments, a concise interpretive paragraph in the Discussion would be sufficient.
2- The work currently cited as reference 17 has since been published. That study reports that cell-surface MT1-MMP expression is a key determinant of invasion in vimentin-positive cells, which fits with the authors’ conclusion that matrix remodeling participates in the vimentin function in collective migration. Please update the citation to the published version and, if appropriate, add one or two sentences in the Discussion to situate the MMP-dependent findings in light of MT1-MMP’s specific role in vimentin-positive invasion.

Version 2:

Reviewer comments:

Reviewer #1

(Remarks to the Author)
The authors have done a nice job answering all questions and revising the manuscript.

Open Access This Peer Review File is licensed under a Creative Commons Attribution 4.0 International License, which permits use, sharing, adaptation, distribution and reproduction in any medium or format, as long as you give appropriate credit to the original author(s) and the source, provide a link to the Creative Commons license, and indicate if changes were made.
In cases where reviewers are anonymous, credit should be given to 'Anonymous Referee' and the source.
The images or other third party material in this Peer Review File are included in the article’s Creative Commons license, unless indicated otherwise in a credit line to the material. If material is not included in the article’s Creative Commons license and your intended use is not permitted by statutory regulation or exceeds the permitted use, you will need to obtain permission directly from the copyright holder.
To view a copy of this license, visit https://creativecommons.org/licenses/by/4.0/

Reviewer #1 (Remarks to the Author):

This manuscript describes migration of mouse fibroblasts from a spheroid into a collagen gel. The authors were interested primarily in the effects of the intermediate filament vimentin, and they compared results for wildtype fibroblasts (which expressed vimentin) and fibroblasts from vimentin knockout mice. The main observations were that cells expressing vimentin showed more migration/invasion into the collagen gel and these cells reorganized and deformed the matrix more than vimentin null cells. The authors attributed these differences to a combination of MMP activity and fluidity within the spheroid, which was assessed through a computational 3D vertex model. The findings on effects of vimentin will likely be of interest to the field, but there are many open questions that the authors must address about this manuscript. The questions are listed below, approximately in order of importance.

1. Details of the 3D vertex model are not given in the manuscript. The manuscript cites a prior study, but the essential components of the model need to be included in the present manuscript. The results section should briefly describe the key components of the model. For example, are the cells given elasticity associated with volume and surface area as is common in a 3D vertex model? Are the fibers given elasticity in response to both stretching and bending, as is common in models simulating fibrous materials? How are the active cell forces simulated? How are the cells connected to the fibers? (Lines 303-304 make a specific claim about cells pulling on fibers, so the connection between cells and fibers seems to be important.) Full details of the model should be included in the methods section or supplemental information. These details are essential to understand the relevance and findings of the computational model, and, since they are not included in the first version of the manuscript, it is possible that new questions about the model will arise in a future round of review.

Thank you for the question and comment. The spheroid is modelled using a 3D vertex model, where the cell's near incompressibility is encoded in a volume spring and the cell's contractility is encoded in a surface area spring. Moreover, cells share interfaces. The collagen matrix is modelled with a semi-flexible polymer network. As for the interaction between the cells and the fibers, a controlled number of surface cells on the spheroid are connected to nodes in the fiber network to the center of the surface face using active linker springs. These are linear springs, where the natural length of the spring decreases at a constant rate until reaching a threshold. This decrease captures the molecular clutch mechanism for integrins. If a surface cell rearranges with other cells and moves into the bulk of the spheroid, the active linker spring is removed and a new active linker spring is added. We have now added a major description of the model to Methods SI page 7.

'"

Simulation Model. The spheroid is represented computationally as a 3D vertex model, described by the energy functional:

$$E_{VM} = K_V \sum_j (V_j - V_0)^2 + K_S \sum_j (S_j - S_0)^2 + \Gamma \sum_\alpha \delta_{\alpha,\beta} A_\alpha$$

where V_j and S_j represent the volume and surface area of the j-th cell, respectively. K_v and K_s are volume and area stiffness coefficients. Together with the target cell volume V_0 and target cell surface area S_0, these constants capture the cell's bulk elasticity and actomyosin cortex's isotropic contractility, respectively. Finally, the last term sums over the boundary polygons (with areas A_0) of the spheroid, with Γ representing the interfacial tension between the spheroid and the fiber network.

The collagen, constituting the extracellular matrix, is represented using a lattice model for a disordered elastic fiber network. Specifically, the fiber network is initialized by randomly linking nearest neighbors on a FCC lattice with some finite probability. Each node is then split into three phantom nodes so that there is a maximum of four each per phantom node. Connected nearest-neighbor bonds that are initially parallel to the principal axes of the FFC lattice constitute fibers in our model. The energy functional of the fiber network is given by

$$E_{FB} = \frac{k_S}{2} \sum_{\langle ij \rangle} f_{ij} (l_{ij} - l_0)^2 + \frac{K_B}{2} \sum_{m=1}^{3} \sum_{\langle ijk \rangle = \pi_0} f_{ij} f_{jk} (\theta_{ijk}^m - \pi)^2$$

where, $f_{ij} = 1$ if a bond is occupied and $f_{ij} = 0$ if not. The length of the bond <*ij*> is l_{ij}. These bonds have stiffness K_S and natural length l_0. Furthermore, θ_{ijk}^m represents the angle between nearest neighbor bonds that are crosslinked by the same phantom node with phantom node index m.

A fixed number of surface polygons on surface cells of the spheroid are connected to nearby fiber ends on the network via active linker springs. These linker springs model focal adhesions between the cells and the collagen matrix. At the beginning of the simulation, the natural lengths of these linker springs shrink at a constant rate to mimic the molecular clutch mechanism for focal adhesions.

Cell rearrangements are a central component of the model because they allow a confluent tissue to flow and reorganize while maintaining its integrity. In two dimensions, these neighbor-exchange motions are known as T1 transitions, and in three dimensions they take the form of reconnection events. Prior work has established algorithms for such 3D reconnections, focusing

on when short edges transition into triangular faces and vice versa once their maximum edge length falls below a threshold. To ensure that these events represent physically valid cell rearrangements, additional constraints are imposed: the moves must be geometrically, energetically, and topologically reversible. Recent refinements include preventing two polyhedral cells from simultaneously sharing multiple faces, which would violate topological reversibility.

Beyond changes in cell connectivity, the model evolves through overdamped Brownian dynamics applied to each cell vertex. The motion of any vertex is described by a balance of conservative forces - arising from area and volume constraints, cell–cell interactions, interfacial tension between the spheroid and surrounding fiber network, and active linker springs - and stochastic forces representing internal cellular fluctuations. These fluctuations are encoded through an effective temperature, which is small compared with the deterministic force contributions but helps the system explore the underlying energy landscape. Vertex positions are updated using the Euler–Maruyama scheme, while the fiber-network nodes evolve via overdamped Euler integration with no active noise term.

Finally, to construct the initial geometry, a bulk Voronoi tessellation is generated, from which a spherical cluster of cells (a "spheroid") is extracted. A surrounding fiber network is carved out to accommodate the spheroid, and active linker springs are assigned along a chosen fraction of boundary faces. After a specified relaxation period, these springs begin contracting toward their reduced target length, driving the coupled evolution of the tissue and fiber network. Simulation parameters are selected based on earlier fiber-network studies and computational efficiency, with conversions to biophysical scales provided (e.g., one simulation length unit ≈ 10 µm, one simulation time unit ≈ 0.144 s). For clarity, all results are reported in dimensionless form.
'''

2. Lines 314-315 suggest that a more fluid-like spheroid causes more deformation and rearrangement on the surrounding collagen. How, specifically, does this occur in the computational model? The results in the present manuscript do not convey how this might be possible. Is there experimental evidence to support how the computational model treats the interaction between cells and fibers?

Thank you for the question. The deformation and rearrangement of the fiber network (which computationally models the collagen) is a consequence of a positive feedback mechanism between the fiber network and the spheroid. Namely, deformations in the spheroid apply strains on the fiber network, which remodels accordingly. The remodeled fiber network, in turn, changes the profile of applied stress on the spheroid. Spheroids with greater fluidity are susceptible to larger shape deformations (via increased reconnection events for cells within the spheroid itself) under such variations in applied stress. Hence, fluid-like spheroids enhance the remodeling of the surrounding collagen. The enhancement of remodeling with fluid-like spheroids in the computational model has been studied in further detail in previous work (https://pubmed.ncbi.nlm.nih.gov/40656459/ Tao Zhang, et al). The coupling between the

spheroid and the fiber network, which facilitates this positive feedback, is modelled using active linker springs, again, acting as molecular clutches.
We have edited the main text to address this point in the second paragraph of the "Increased tissue fluidity enhances collagen remodeling" section in the result on page 12.

3. In Fig. 6d, the results from experiments and computational model are quite different. Specifically, the blue curve is slightly above the red curve for experiments, but for model, the blue is very substantially above red, especially for moderate and large distances from the center, which is notably different from the experimental data. Can model parameters be further tuned for better match? If no, is the model a relevant one for comparing to these experiments?

We have made some corrections to this graph (discussed further in response to question 7): including a normalization factor in the modeling plot and accounting for overall drift of the spheroid. We now observe that the displacement for the fluid displacement is smaller in the computational model as compared to the experiments. As the computational model does not include the cell elongation and other effects such as fiber breaking, we expected differences between the result of the experimental data and the computational model. Note that the difference between the fluid-like spheroid and solid-like spheroid curves can be further reduced (as shown below – Figure S4e) by reducing the time interval in which the displacements are measured. Finally, a smaller difference in s0 between the two graphs can also reduce this difference.

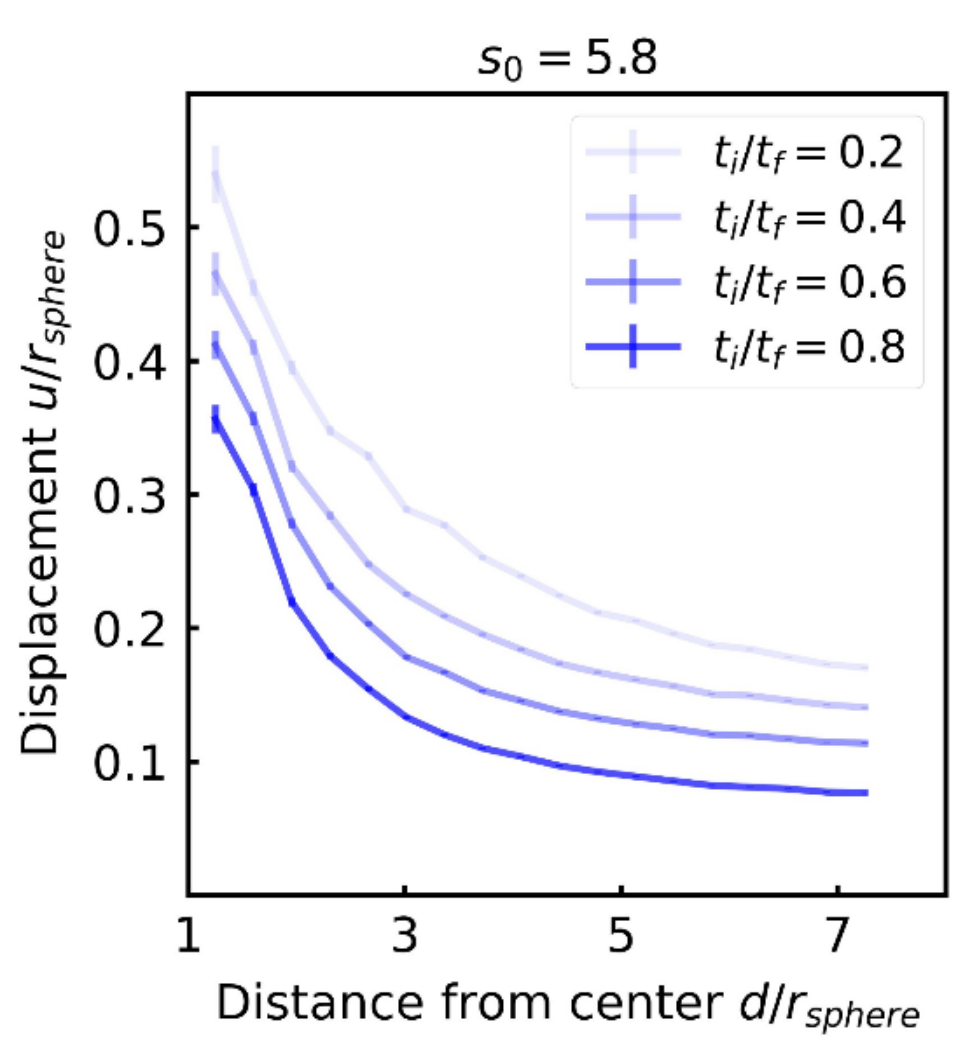

4. Additional clarity in writing is needed when describing matrix degradation. The last sentence of the abstract refers to "MMP transportation," but the authors didn't measure MMP production or transport and didn't show those were affected by vimentin; instead, they showed that MMPs were important for invasion. In the first sentence of conclusion, the authors state, "our experimental results motivate a physical mechanism in which vimentin-mediated collagen degradation...," but the data didn't show that the degradation was mediated or affected by

vimentin; instead, the data showed more invasion for vimentin-expressing cells and that degradation depended on MMPs. One possible explanation for these observations is that vimentin enhances degradation, but this was not shown by the data—in fact, the main support for the effects of vimentin on MMP activity comes from citing prior literature, and not the authors' experimental measurements. The authors should revise the text to clarify what can be concluded based on their own experiments and what can be inferred by comparing to prior literature. The authors are encouraged to read the other sections of the manuscript (such as results and paragraph 4 of discussion) to ensure accuracy of description of the results from this manuscript and a clear distinction made between conclusions that can be drawn directly from this manuscript and conclusions made by integrating results from this study and others.

Thank you for the suggestion, we have changed the wording from "MMP transportation" to "collagen remodeling" and remove mentions of MMP-transportation in the conclusion. The result section no longer contains any mentions of MMP transportation. We have also made it clear in the discussion that the Vimentin-related transportation of MMP is the result cited directly from reference 22.

5. The authors use the words "traction," "traction force microscopy," and "contractility" (with units of force), but the methods used are questionable for quantifying cell tractions or forces. The constitutive relationship used for the collagen gel is not stated, and it's still an open question in the field as to what constitutive relationship to use for fibrous materials. The data reporting contractility also used 2D imaging, despite this being a 3D system, which creates problems in computing the tractions (and, in turn, the contractility shown in Fig. 3c). The approach to quantify contractility assumes a sphere, but for wildtype cells, there are invading strands, meaning the system is not spherically symmetric. For the purposes of this study, showing bead displacements is sufficient. The authors should keep the displacement data but remove the contractility data and remove the word traction from the manuscript.

Thank you very much for the comment. We have updated methods section for contractility calculation and restricted the figure 3c to 8 hours (where no cell breakout has occurred). Updated section now includes:

'''

The material simulation was perform using saenopy package (included in jointforce) which is a semi-affine fibers material model. The fibers are modeled to show different behavior in three strain regimes: under compression, fibers buckle showing exponential decay of the stiffness; under small strains, fibers are under linear stiffness regime; and for larger strain, fibers exhibit strain stiffening with exponential increase of the stiffness. The stiffness k_0 is thus given by

$$w''(\varepsilon) = k_0 \cdot \begin{cases} e^{\frac{\varepsilon}{d_0}} & if \ -1 \leq \varepsilon < 0 \ (buckling) \\ 1 & if \ 0 \leq \varepsilon < \varepsilon_s \ (linear) \\ e^{\frac{\varepsilon - \varepsilon_s}{d_s}} & if \ \varepsilon_s \leq \varepsilon \ (stiffening) \end{cases}$$

where the buckling exponent is denoted as d_0, the stiffening threshold as ε_s, the stiffening exponent as d_s and the energy function as $w''(\varepsilon)$.

'''

In addition, we have replaced "traction" with "effective contractility" for better representation that the force is approximately found using Christoph Mark's model.

6. Fig. 5d shows interesting results of cells migrating into pre-made tracks, but the images are low contrast and it is difficult to see the cells. Can the authors provide higher contrast images? Additionally, can the authors quantify the results, e.g., distance of migration or something similar?

We have changed the Vimentin-null image in Figure 5 to show the cells better. On average, 2-4 cells escaped approximately 100 um into the channels for both cell types. We have added this information to the main text under "Vimentin-mediated migration is MMP-dependent" result section on page 10.

7. In Fig. 6d, it is unclear how the displacement data is normalized. The figure axes say u/r_0 (presumably r_0 is the initial spheroid radius) but values are greater than 1. If the displacement is inward, a value of 1 would mean that the displacement is equal to the spheroid radius, i.e., the spheroid has collapsed to a point. Values greater than 1 shouldn't be possible. Can the authors check the normalization and define it in the caption?

Thank you for the question and for pointing out the discrepancy in the normalization on the graph. Yes r_0 does represent the initial radius of the spheroid. For clarity, we have changed this notation to r_sphere and defined it in the figure caption. This quantity is used to set a length scale for the displacements of the fiber nodes.
As mentioned previously, we have corrected the graph for normalization and drift.

8. In Fig. 7, it looks like the initial size of the spheroid is different in the sample images in 7b. I see 7c normalizes by initial area, but if the initial size is drastically different, as it is here, there might be fewer cells that can invade, which would in turn limit the total spheroid area. Are the initial sizes substantially different?

The initial sizes are not substantially different between population on average. We have updated figure 7 to include initial-final image pair to further clarify this.

9. On line 145, the authors state that the maps of displacement field correlate with regions of invading strands of cells. The image shown in Fig. 3 shows only the displacement maps. Not the cells. The authors should add images of the cells for this experiment so that the data support the correlation between cell invasion and locations of displacements.

We have moved the aggregated displacement map to Fig S7a and replaced it with a snapshot of the displacement map with the spheroid.

10. Line 176-177 says "Our results thus far demonstrate that vimentin plays an important role in

collective migration of fibroblast cells through collagen networks." This wording is not quite accurate. The data show that cells with vimentin migrate more than cells without vimentin, i.e., migration correlates with vimentin. But the wording implies that vimentin is the cause. The authors should revise this wording to be more precise in describing the results.

We have changed "plays an important role in collective migration" to "correlates with the invasiveness".

11. Line 291 refers to a "time point sufficiently large enough to allow for both the cells and fibers to deform…." Can the authors provide more detail on how the time point was determined? The following text refers to a "new equilibrium"—does this text about time have something to do with the computational solver or is it a meaningful representation of time in the experiments? The text here also refers to supplemental information, but the supplemental information does not appear to have any information about the time point selected to show.

Thank you for the question. Since the simulation starts with randomly generated configurations for the spheroid and fiber network, an appropriate delayed initial time point is chosen for taking measurements. For all samples, the average displacement and resulting densification of fiber nodes are measured with respect to the states from this time, up to the final states of the samples. Aside from avoiding the initial random configurations, this initial time point is also chosen to be after the active contraction of the linker springs is complete, with these active pulling forces having caused rearrangements in the spheroid and fiber network. Further dynamics of the system past this initial time point is due overdamped motion, with added random activity in the spheroid. We have included a plot of displacement graphs with different choices of time points in the (shown above in addressing question 3 and Figure S4e). We have added the text to the Simulation Data section in the Supplemental Information.

12. Starting in paragraph 2 of the discussion, the authors talk about how the findings are surprising. One finding not included in this section is that vimentin correlates with greater cell aspect ratio. Isn't this finding an expected one, given that vimentin is often correlated with mesenchymal phenotypes?

Thank you for the comment. We would expect a more elongated nuclear/cell shape for mesenchymal cells. Thus, we have removed the word 'unexpected' regarding the finding. We have cited studies that show vimentin is correlated with cell elongation in paragraph 5 of the discussion.

13. The authors use the word "polar" or "polarized" in the abstract, results, and discussion. The word polar implies a vector quantity with a front and back. The experiments and computational model do not quantify a front or back of the cell; instead they quantify an aspect ratio. Instead of using the word "polar," wording that conveys aspect ratio would be more accurate.

Thank you for the suggestion, we have changed the wording from polarized/polarization to elongated/elongation throughout the result and discussion.

14. The last sentence of the abstract mentions that vimentin has an effect of "enhancing migratory persistence," but the authors didn't quantify migratory persistence in this manuscript. They quantified the total amount of invasion, but not how persistent the migration was. The wording should be updated for more accuracy.

We have changed "migratory persistence" to "collective cell migration"

15. In the images of fibers measured by confocal reflectance in Fig. 2, the contrast is low, and it is difficult to see alignment and density of the fibers. Can the authors provide higher contrast images in the main text?

We have updated the image with higher contrast level for the collagen reflection channel.

16. Text near line 295 refers to collagen strain, but the figures show only displacement. Text about strain should be removed.

We have changed the text to be collagen compaction and reference to figure S4c.

17. In Fig. 1c, the resolution of the images is low. It is difficult to see the localization of actin and vimentin in the cells. Can the authors provide higher resolution images?

We have added a zoomed in inset in Figure 1C to show better actin/vimentin fluorescent (image shown below). Since the spheroids are stained while still embedded in the collagen gel, the vimentin/actin channel does not show up as well as DAPI.

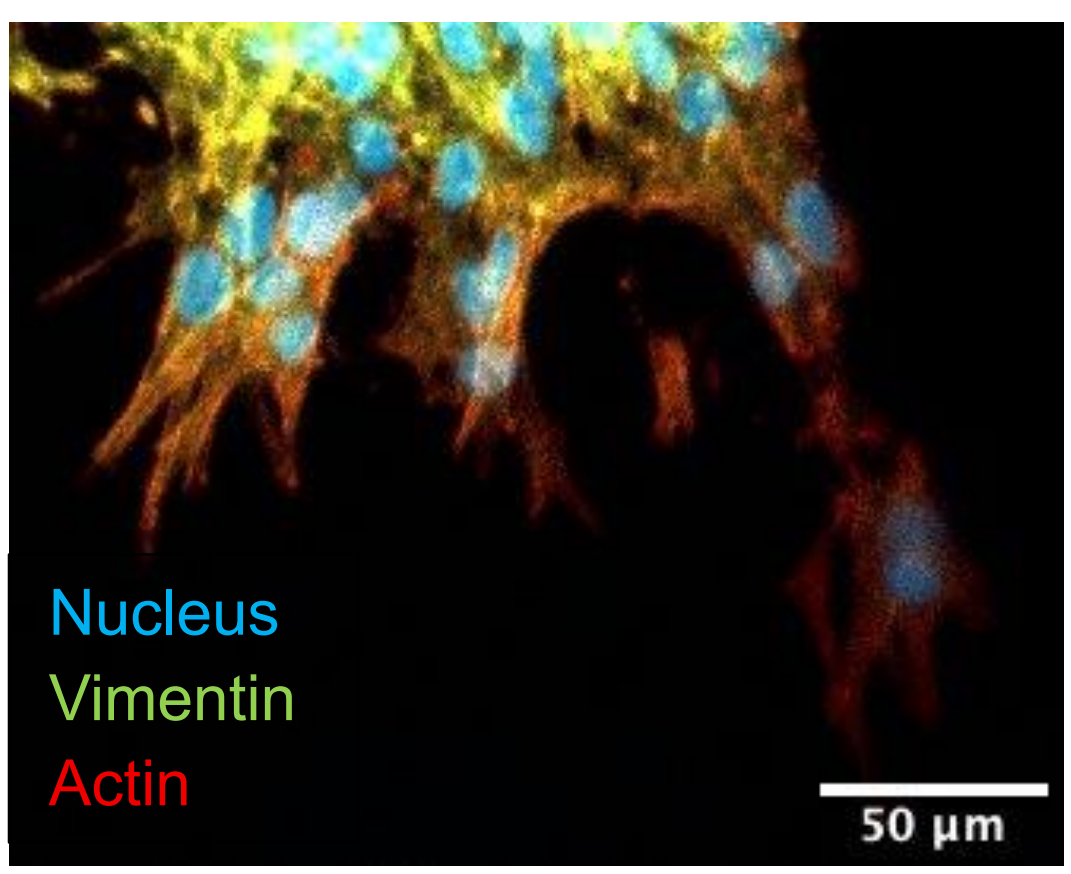

18. The caption to Fig 6c says that the results show nucleus aspect ratio, but the results from the model show cell aspect ratio, not nucleus aspect ratio.

We have fixed the caption to clearly indicate nucleus aspect ratio for experiment and cell aspect ratio for model.

19. Fig 2b has two * symbols; it appears only one is needed for a statistical comparison. Is one an error?

We have fixed the typo, thank you.

20. Line 251 has a typo: “spheroid” should be “spheroids”

We have fixed the typo.

21. Figure 8 does not appear to be mentioned in the main text.

We have now referenced to Fig 8 in the conclusion.

Reviewer #2 (Remarks to the Author):

In this manuscript, Minh Tri Ho Thanh and colleagues investigate the role of vimentin in fibroblast invasion using in vitro 3D collagen models. They compare wild-type and vimentin-null mouse embryonic fibroblasts (mEFs) to assess their capacity for collagen matrix invasion, collagen remodeling, traction force generation, and spheroid fluidity. Their results indicate that loss of vimentin impairs MMP-dependent invasion, decreases collagen remodeling and traction forces, and alters the mechanical properties of spheroids. Interestingly, they also suggest that vimentin-deficient fibroblasts are less effective in promoting adenocarcinoma cell invasion. While the role of vimentin in promoting cell migration and invasion is well established, the manuscript presents some novel and potentially impactful findings—particularly those related to spheroid fluidization and the cooperative invasion of cancer cells. However, most of the results are correlative and mechanistic aspects remain unclear. The study also lacks essential experimental controls, such as rescue experiments and siRNA-mediated knockdowns, which limits the strength of the conclusions.

Major Concerns

1- Lack of validation via complementary approaches

The study relies exclusively on vimentin-null mEFs. The authors should confirm their findings using siRNA-mediated knockdown in wild-type cells and rescue experiments. While Figure 4 presents data using a mutant vimentin unable to form filaments (ULF), it remains critical to demonstrate that re-expression of wild-type vimentin restores invasion capacity, contractility, fluidization.

Thank you very much for the comment, we have performed new experiments with both Vimentin-rescue and Vimentin-knockdown cell line. The result is shown in Figure S5.

2- Weak mechanistic insight
The authors propose that impaired invasion results from increased contractility leading to decreased fluidity and altered matrix remodeling. However, these hypotheses remain untested. Multiple alternative explanations should be considered and experimentally addressed:

- Does vimentin expression affect cell proliferation or survival within spheroids?

There is not a proliferation defect reported for these cells in standard cell culture conditions. There is also no standard way to directly measure proliferation in the cell spheroids. In wound healing assays, Eriksson's group [PMID: 27466403] showed that vimentin affects the proliferation rate and survival rate starting on day 2. Based on our data, we have already seen a difference in cell migration after 24 hours so the increased in invasiveness is not expected due to purely higher cell proliferation or survival. We have made a note of this in paragraph 8 of the Discusion on page 16.

- Are cell adhesion or integrin expression altered in vimentin-null cells?

There are differences in the adhesion found by Zofia et. al. [PMID: 33558312] when these mEFs are cultured on collagen-coated glass slides. However, since the cells are in a physiologically soft collagen gel, we performed new experiments, in which cells were spread on soft collagen-coated polyacrylamide gel (500 Pa). Adhesion data shows no differences in paxillin expression (images below – Fig S7b). The difference between gel and glass is mainly due to the focal adhesion are much more matured when the cells are fully spread out on glass compared to very soft gel.

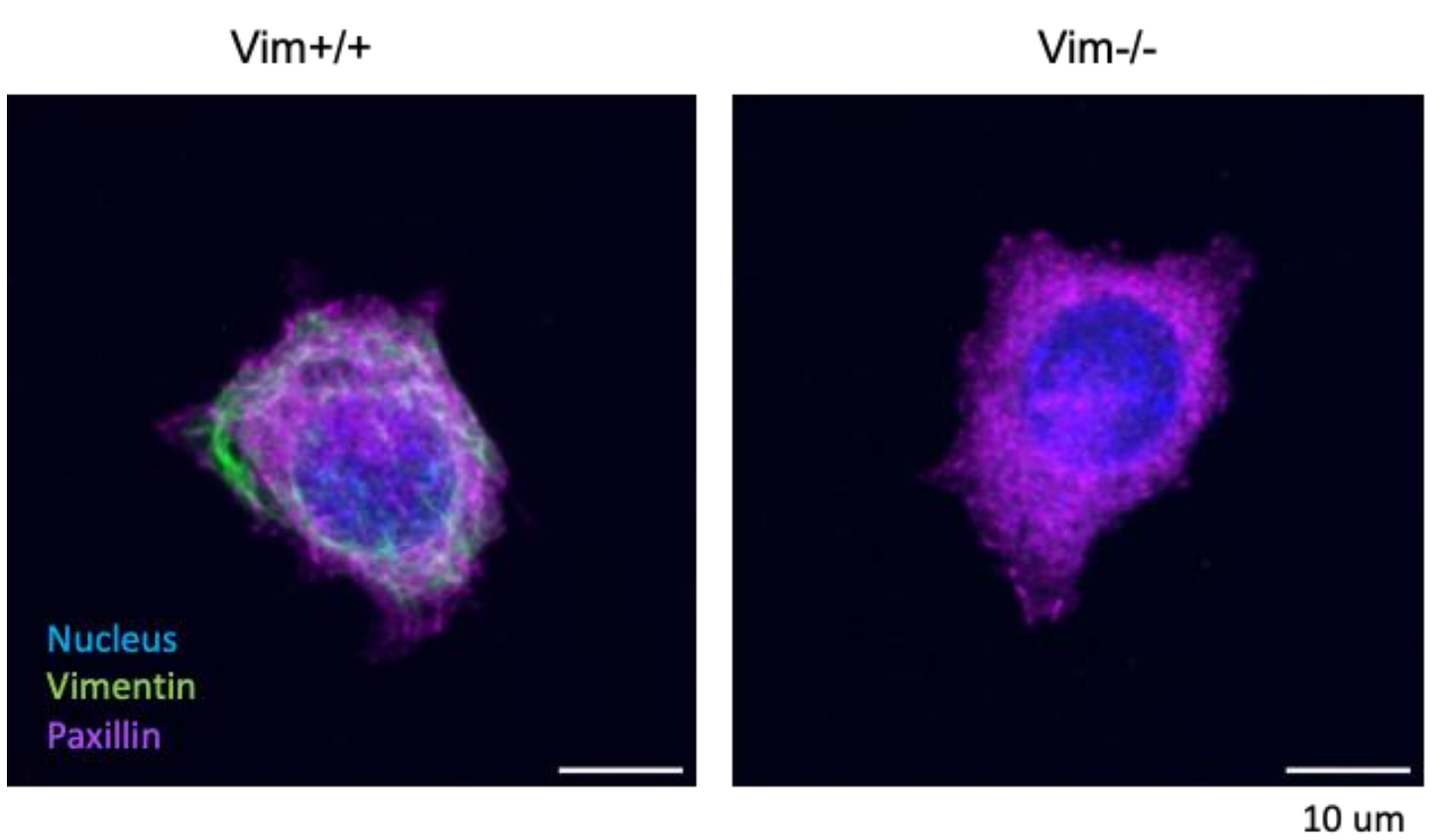

For cell-cell adhesion, we stained for N-cadherin (cells on collagen-coated glass) and we could not observe any difference in fluorescent intensity (images shown below – Fig S7c). It has been shown before in rat embryo fibroblasts that N-Cad does not colocalize with VIF [PMID: 11809840] and N-cad expression is the same in both Wild-type and Vim-/- mouse dermal fibroblasts supernatant media [PMID: 27466403].

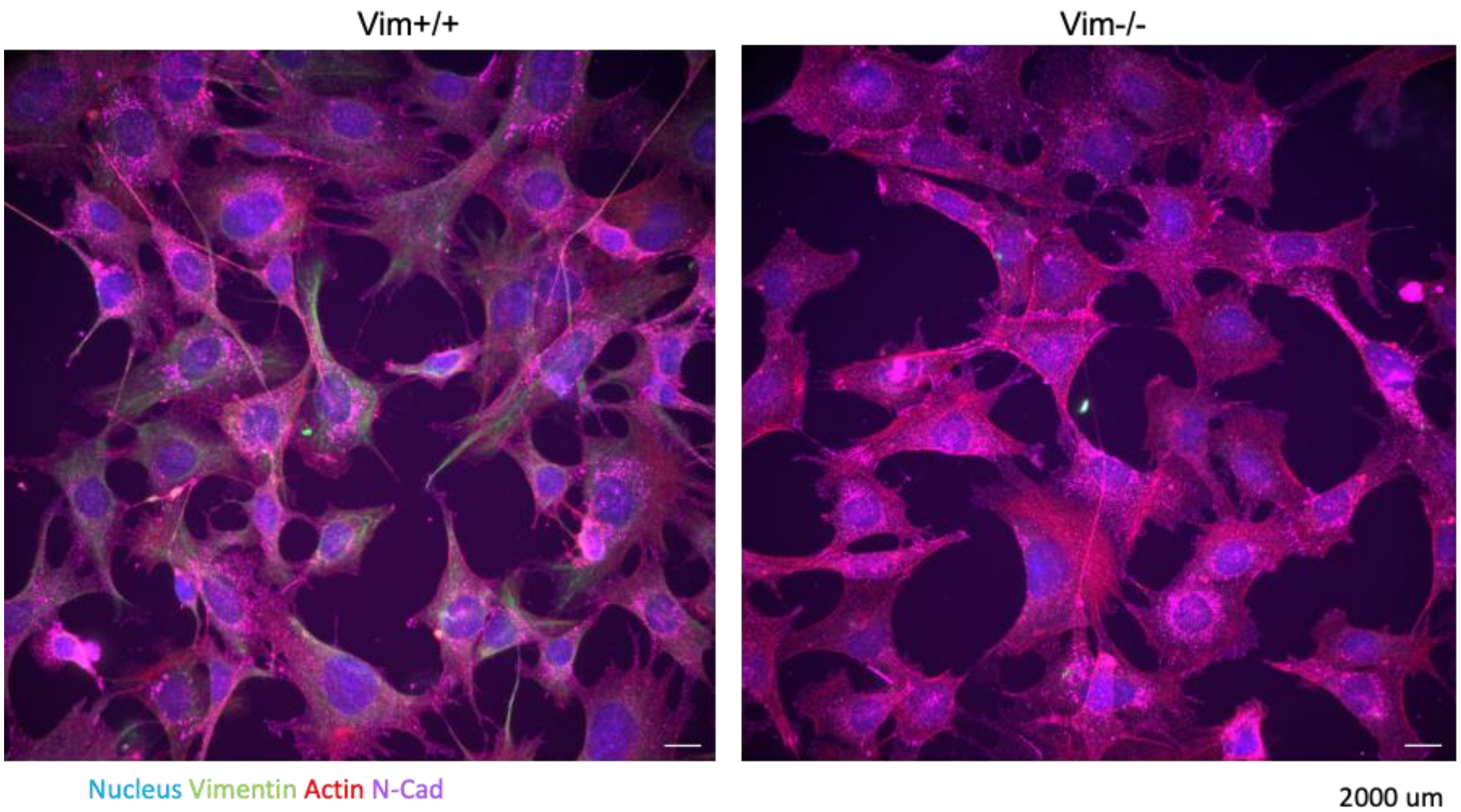

We have added images and citations to paragraph 6 in the Discussion on page 16, where we have previously discussed about cell-matrix adhesion and cell-cell adhesion assumptions.

- Given that invasion appears MMP-dependent and vimentin has been linked to MT1-MMP secretion, is MT1-MMP expression or localization altered?

Zofia et al. [PMID: 37440280] found that the expression level of MT1-MMP is not lower in Vim-null mEFs cultured in collagen gel, however, depleted Vim lower the secreted MT1-MMP and altered the localization of MT1-MMP in the plasma membrane. The text has been edited to address this point in paragraph 4 of the Discussion on page 15.

- Is the reduction in spheroid fluidity (cell/nuclear shape) a cause or a consequence of impaired invasion?

We believe that the change in fluidity is the cause of impaired invasion as it occurs preinvasion (Figure 6). We modified the text to make this point clear in the last paragraph of "Increased tissue fluidity enhances collagen remodeling" section in the result on page 12.

- Are decreased contractility and matrix alignment causes or a consequences of impaired invasion? Does mild inhibition of contractility reduce invasion in wild-type cells?

Decreased contractility and matrix alignment is one of the causes of impaired invasion as we observed lower contractility in vimentin-null spheroids preinvasion.

Mild inhibition reduced invasiveness of the wild-type spheroid. We performed a Cytochalasin D dose-response treatment of the wild-type spheroid in Figure S5.

Specific concerns

1. Figure 1. Are vimentin-null cells entirely incapable of invading, or is their invasion only delayed? Long-term assays (e.g., >72h) would clarify this.

Vimentin-null cells can invade, image in Figure 1b and Movie S2 show a single strand and cell invasion. We have also added a 72h vimentin-null invasion assay to Figure S5.

2. Figure 2. Can the authors provide representative images of vimentin-null spheroids with protrusions, as seen in ~50% of cases (Figure 5b)? Do these spheroids show any degree of collagen remodeling?

Figure 1B Vimentin null bottom left shows a single spheroid with protrusions. Yes, Compaction above 1 and skewed alignment data shows vim-null can remodel the network, but to a much lesser extent compared to wildtype. Below is the enlarge vimentin-null spheroid in Figure 1b.

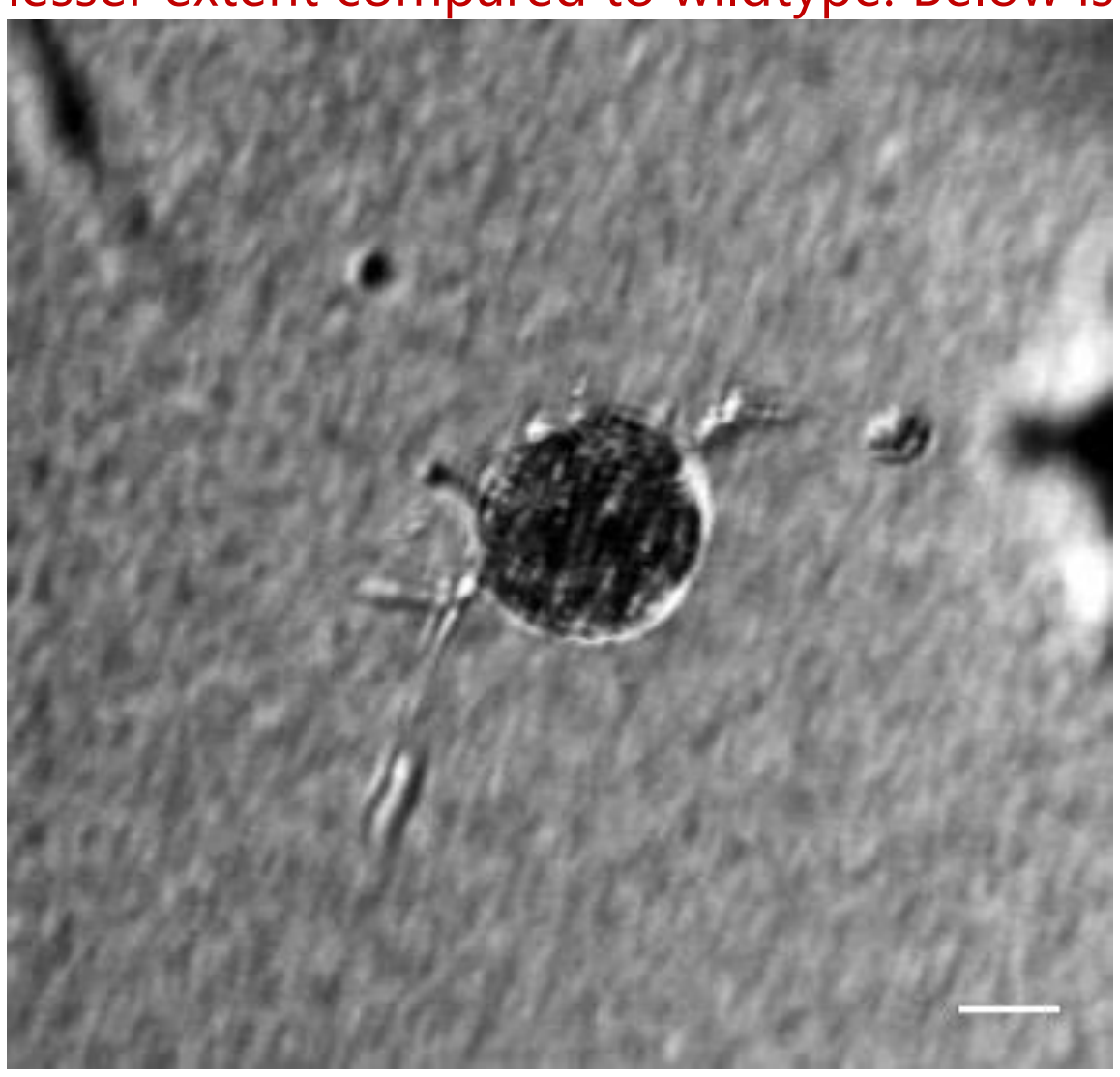

3. The recent study (PMID: 39952241) reporting vimentin-dependent regulation of MT1-MMP in macrophages should be cited and discussed. Do mEFs form podosomes, and are these structures affected by vimentin loss?

As far as we can find from past literature, fibroblasts (and specifically mEFs) typically do not form podosomes. They generally form focal adhesions [PMID: 2800791]. Specific treatment such as TGF-β or the PKC activator [PMID: 37735172], or plating fibroblasts on fluid substratum (where cell-matrix force generation is disabled) [PMID: 24290759] can induce podosomes formation on fibroblasts. Since we did not perform such treatment in our experiment, we do not expect our mEFs to form podosomes. We have now cited and discussed the suggested study in paragraph 4 of the Discussion on page 16.

4. Figure 3a. The spheroid boundary and scale bar should be marked. In the WT condition, it is difficult to identify the spheroid center.

We have moved the aggregated displacement map to Fig S7a and replace it with a snapshot of the displacement map with the spheroid. Scale bar is included.

5. Figure 3b. The x-axis scale is unclear—possibly logarithmic? A clear label and indication of the zero point are needed.

The plot is in in logarithmic scale. We have made it clearer and added a more detailed description in the caption.

6. Figure 4.
a. The analysis at 24h should be extended to 48h for consistency with other figures.
Be consistent with contractility 24h. VimULF timelapse graph in supp

We have corrected this where appropriate. Since the contractility is not accurate after cell breakout, we have limited the contractility to 8 hours. We have now plotted the Vimentin ULF timelapse in figure 4c.

b. Presenting the data as histograms is also inconsistent with the curves used in Figures 1 and 2. Time-course curves would be more informative.

We have plot the Vimentin ULF timelapse in figure 4c now.

c. This figure should include data from vimentin siRNA/shRNA knockdowns and wild-type rescue experiments.

We have repeated the experiment with Vimentin-rescue and Vimentin-knockdown cell line. The result is shown in Figure S5.

d. Figure 4d includes statistical analysis with only two independent replicates, which is insufficient.

We have added a replicated and plot the Vimentin ULF timelapse.

7. Figure 5. The authors should normalize the normalized spheroid spreading area as in the other figures.

We have replotted figure 5A to include the timelapse snap of the spheroid incubated in MMP-inhibitors for better visibility. The normalized spheroid spreading area day by day has now been plotted in Figure S4e

8. Figure 5c, 5d. Laser-Ablated Channel Assay. The qualitative result is compelling but lacks quantification. What proportion of total invading cells enter the channel in each condition? What is the absolute number of cells in channels per condition?

On average, about 2-4 cells escaped into the channels for either cell types. This point is added to the main text on page 10, under "Vimentin-mediated migration is MMP-dependent" section of the result.

9. Figure 6. Conclusions about spheroid fluidity are based on nuclear deformation, whereas fluidity should relate to cell shape. The comparison between experimental nuclear aspect ratios and simulated cell shapes is not well justified. Notably, nuclei (shown in a) appear elongated along the spheroid surface, rather than perpendicular to it, which, to me, contradicts the proposed mechanism where fluidization facilitates invasion.

Fluidity is directly related to the cell shape. Due to low fluorescent signal in membrane staining embedded spheroids combined with low membrane segmentation quality of 3D cell aggregates for several opensource software [ref 70, PMID: 32612752], we have chosen to use nucleus shape instead; as it has been shown that in fibroblasts, nucleus shape and cell shape are highly correlated [PMID: 26210179, 37397244]

We don't know how cell break-up from spheroid emerges. It is not clear that perpendicular alignment decreases cell break-up from spheroid. We propose a more elongated cell is more fluid-like and therefore more likely to contract the ECM network.

10. Figure 7. The description of observations is vague and lacks quantification. Specific questions need to be addressed:

a. Do mEF and MDA-MB cells proliferate at the same rate? Is the 1:2 ratio maintained over time?

mEFs typically have doubling time from 24-48 hours [PMID: 23441817, 30798523] while MDAMB231 has a doubling time of 27-31h [PMID: 23094027, 1768053, 32497154]. In coculture, it is much more complex as it is unclear how fibroblasts enhances/inhibits cancer cells growth and how cancer cells growth activates fibroblasts into CAFs [PMID: 36917949, 36197901].

b. Are both cell types homogeneously mixed, or is there evidence of cell-type-specific segregation?

We have a Celltracker image of the coculture spheroid in Figure S4. We observed that there is no preference for shell-core demixing of mEFs/MDAMB231.

c. Which cells are invading—mEFs, MDA-MBs, or both? Are WT fibroblasts leading cancer cells during invasion?

Our Celltracker image of the coculture spheroid shows that there is no clear leader-follower relationship between mEF and MDAMB231 in our collagen matrix. Our collagen concentration of 1.5mg/ml is a low collagen concentration. Cancer cells demonstrate reduction invasive capabilities at higher collagen concentration ≥ 4mg/ml (https://doi.org/10.1038/s41598-018-30683-4), which we think where fibroblasts/CAFs would play a much more significant role in leading invasion.

d. Could reduced invasion in mixed spheroids with vimentin-null fibroblasts simply result from the large presence of a non-invasive (vimentin null mEF) population?

We have added a 3:1 (MDAMB231:Vim-/-mEF) coculture spheroid experiment to test this hypothesis for our 48-hour experimental time frame. These 3:1 ratio spheroids have similar invasive profile as the MDAMB231 monoculture spheroids (shown in Fig S5).

Reviewer #1 (Remarks to the Author):

The revised manuscript and point-by-point response have addressed most of the comments from the first review, though some open questions still remain. Numbers below correspond to the same numbers in the first review.

1. Question 1 of the first review was to give details about the model. Those details were added to the supplemental information, but some open questions still remain that are important to describe the model effectively:
- It is not clear what is meant by the sentence "Each node is then split into three phantom nodes so that there is a maximum of four each per phantom node." The meaning of a phantom node is not clear, and it is not clear what "four each" is referring to. Is this referring to how fibers connect to nodes? This should be revised to give specific details, because it is well established that the number of fibers that connect at each node (often referred to as the "coordination number") is a very important factor in the behavior of matrix materials.
- Equations are given for the simulations, but the simulation parameters are not stated. Specific parameters are K_B, k_s, K_v, V_0, K_s, S_0, and Lambda. The text says the parameters are based on prior studies, but no citations are given. It is necessary to state the simulation parameters and demonstrate (with citations) that they're within the range of prior studies.

Thank you for the question/comment. The meaning of the phantom node sentence is that each node is split into 3 sub-nodes in which a maximum of four edges representing two fibers are crosslinked. We have used the established terminology of "phantom nodes". However, if such terminology is unclear then we can modify it to sub-nodes. We have opted for the latter in the revised manuscript given the confusion. Here is our revised sentence(s) in the manuscript:

"Each original node is replaced by three sub-nodes ("phantom nodes"), such that each sub-node connects to at most four edges (corresponding to two fibers, each contributing two segments). This construction effectively limits the local coordination number to ≤4."

We have also now included simulation parameters in the revised supplementary so that the manuscript is now more self-contained. A citation to our prior work, which contains the reasoning behind the chosen parameters, is now also provided in that section of the manuscript as well.

3. Question 3 was about comparison between experiments and modeling of matrix displacements over distance from the center of the spheroid. The updated Fig 6 still does not show model results that convincingly capture the experiments. In the updated figure 6D, the red line for the model is still notably different than in the experiments (in the model, it's still notably below the blue curve). The authors suggest that a different time step and/or

other values of S_0 can improve the comparison--this should be done with updated model results presented in main text OR the main text should acknowledge that there is quantitative disagreement between experiments and model and also describe why/how the qualitative trends in from the model are sufficient to interpret the experiments.

Thank you for this comment. We agree that the current model does not achieve a quantitative agreement with the experimental displacement profiles. Our goal in constructing the computational model is not to achieve precise quantitative agreement through parameter tuning, but rather to develop a minimal, mechanistic framework that captures the essential physical processes governing matrix deformation. While it is always possible to adjust parameters (e.g., time step or s_0) to improve agreement, doing so without incorporating additional biological mechanisms risks overfitting and obscuring the underlying physics.
Importantly, the model does reproduce the qualitative trends observed experimentally, including the spatial decay of matrix displacements away from the spheroid and the overall form of the displacement profiles. These features allow us to interpret how mechanical interactions between cells and the extracellular matrix give rise to long-range remodeling. The remaining quantitative discrepancy likely arises from biological processes not currently included in the model. In particular, cells in experiments actively degrade and remodel the collagen network over time, which would affect local network stiffness and alter the displacement profile. Because collagen degradation is not incorporated in the present model, a fit for cell target shape index s_0 is not expected to match the experimental displacement profile at every measured distance from the spheroid.
We therefore interpret the model predictions as providing a generic comparison between rigid and fluid spheroid-induced matrix deformations, in the absence of active biochemical remodeling. This perspective allows the model to serve as a baseline for identifying which additional mechanisms—such as collagen degradation—are necessary to achieve quantitative agreement. We have revised the manuscript to (i) clarify the minimal nature of our model and that the missing biological processes can result in quantitative differences from, but qualitative agreement with, the experimental data (ii) explicitly acknowledge the quantitative differences, (iii) clarify the qualitative agreement.

5. Question 5 was about how tractions were calculated from the experiments. It is good that the language has been changed from “traction” to “contraction” and it is good that details have been added about the methods. Two remaining points are:
- The description of the method talks about "fibers" but the approach is a continuum approach, meaning it doesn't actually account for single fibers. (The term "semi-affine" has been used by some prior manuscripts and gives the impression that this approach accounts for individual fibers, but it does not; the method originally developed by Steinwachs et al. uses a continuum approach.) Given that spheroids are much larger than the fiber lengths, a continuum approach is probably fine, but the authors should revise the text in the methods section describing the method to clarify that this method treats the matrix as a continuum.

- The model has various constants. According to the documentation on Github, there are 4:
K_0: the linear stiffness (in Pa)
D_0: the rate of stiffness variation during fiber buckling
L_S: the onset strain for strain stiffening
D_S: the rate of stiffness variation during strain stiffening.
The constants used need to be stated.

We have added the following sentences to the description of the material model for clarity and cite Steinwachs et al.

“The material model (not affine material) assumes the biopolymer network as a mechanical continuum with some affine properties, which is approximated by a mean-field description. Individual fibers are not simulated; the model uses a mean-field approximation to capture their collective mechanical effect”

The parameters were included in figure S3. We have added them to the end of the effective contractility method for better visibility.

Reviewer #2 (Remarks to the Author):

The authors have satisfactorily and substantively addressed nearly all rmy previous concerns. The revision presents a coherent, well supported story linking vimentin to collective migration through MMP dependent remodeling, and spheroid fluidization. I just have two minor points which I think should be addressed before publication.

1- In the rescue experiments, re-expressed vimentin in vimentin-depleted mEFs appears as a fragmented/patchy network rather than a typical extended filament meshwork. Yet, those cells recover the “less-fluidized” phenotype and regain aspects of the vimentin-dependent migratory program. It would strengthen the manuscript to explicitly discuss how a fragmented vimentin network could mechanistically restore function. Can it be a mechanical function or does it have a signaling role. If feasible within scope, a brief quantification (added to Methods/Supplement) would help: e.g., filament length in the rescue lines, and whether these correlate with rescued contractility/MMP activity. In absence of new experiments, a concise interpretive paragraph in the Discussion would be sufficient.

We have rechecked our stained images and only see fragmented vimentin filament in the Vimentin knockdown cells using siRNA. Below are the enlarged images of the filament network of the Vim+/+, Rescue, and Knockdown cells. We have included the Vim+/+ stained image to Figure 5S for better comparison.

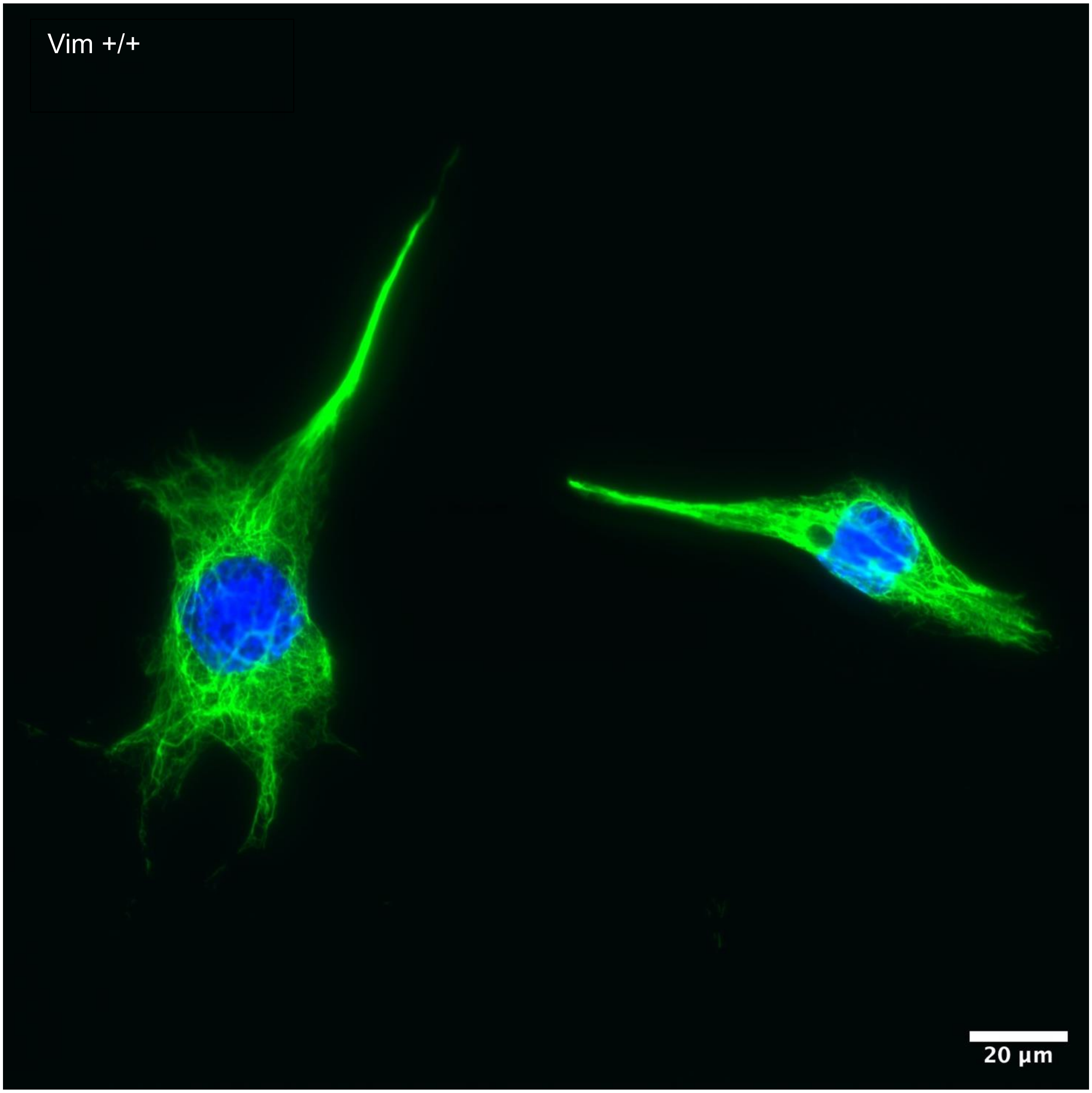

Nucleus
Vimentin

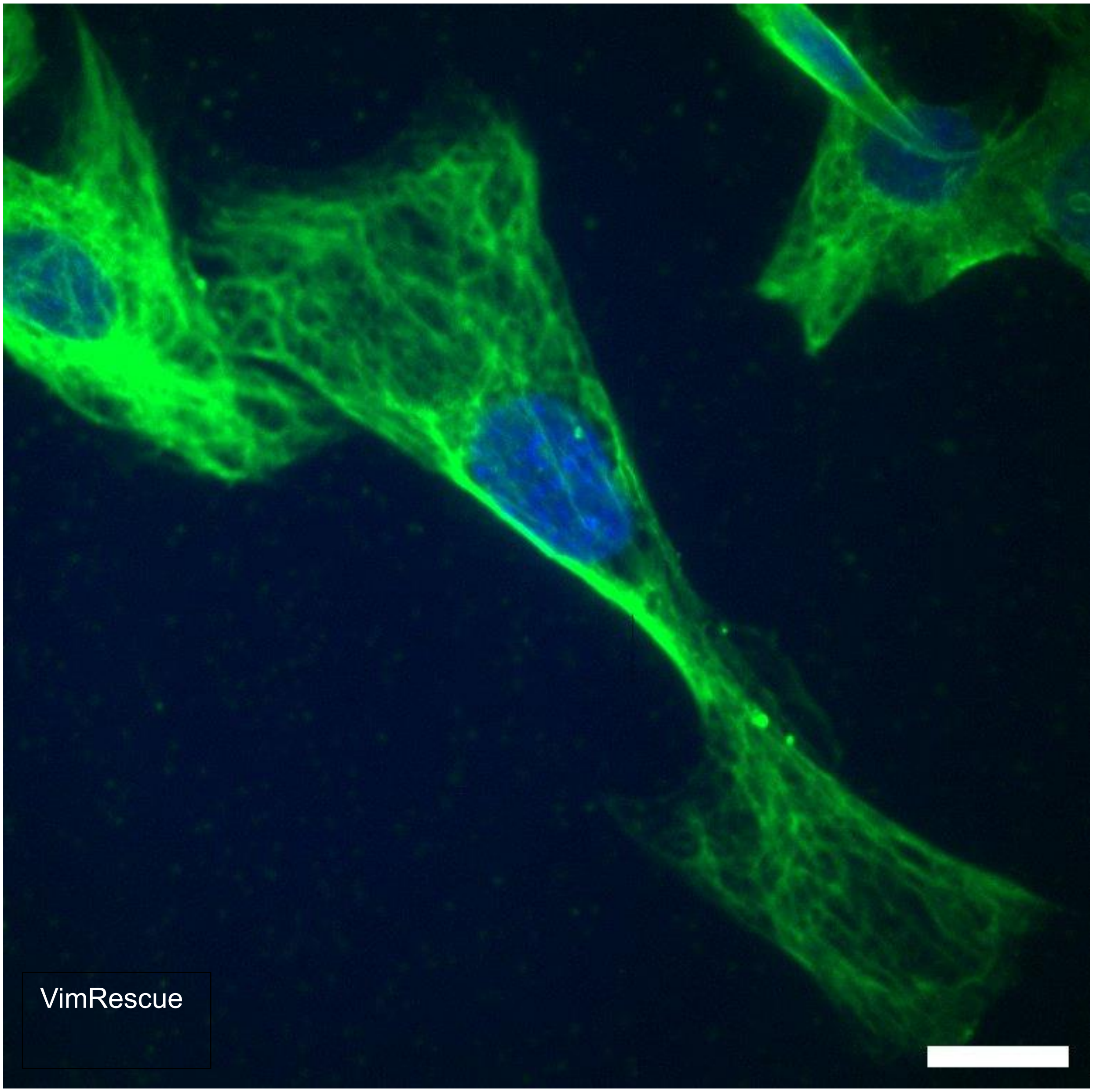

Nucleus
Vimentin

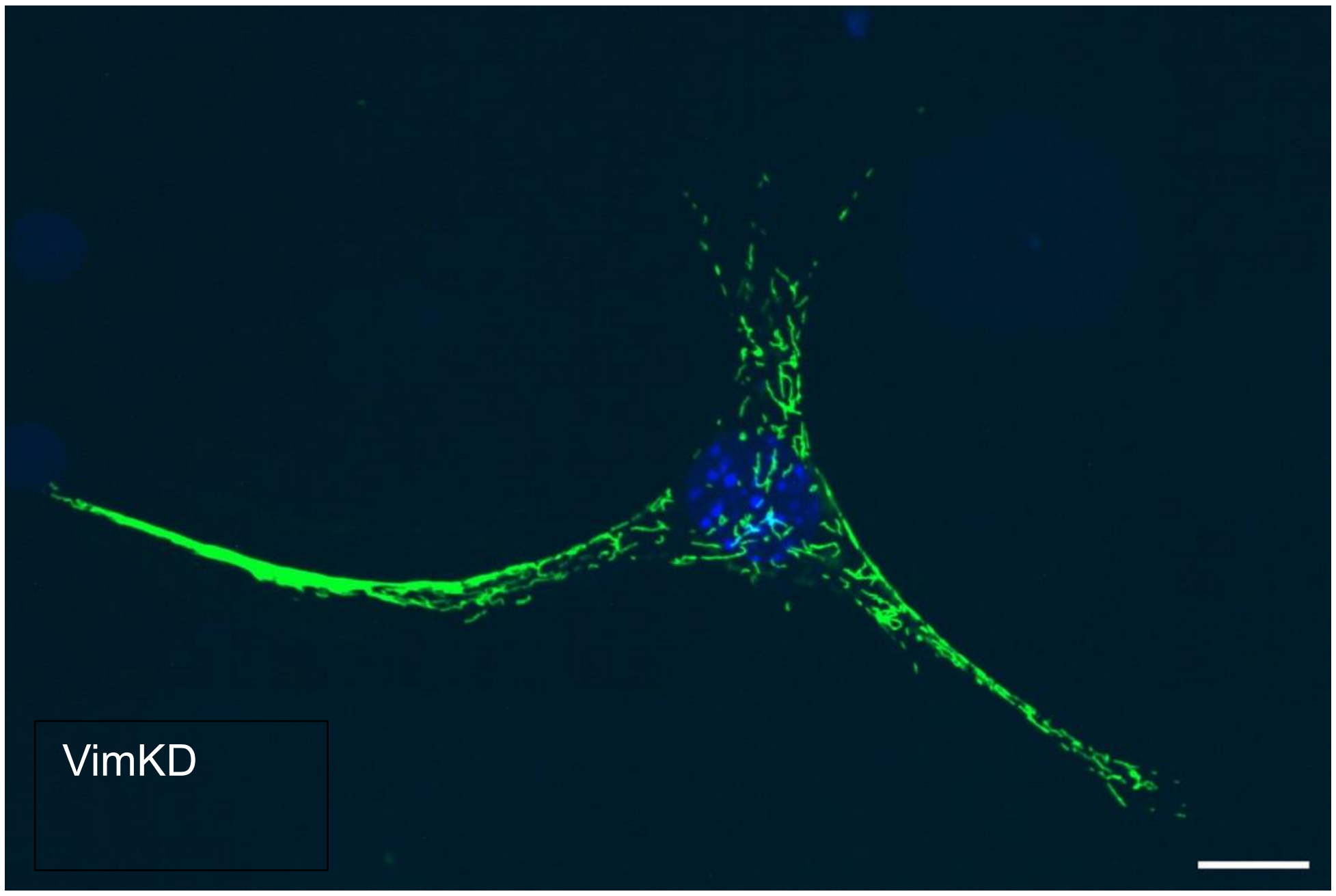

Nucleus
Vimentin

2- The work currently cited as reference 17 has since been published. That study reports that cell-surface MT1-MMP expression is a key determinant of invasion in vimentin-positive cells, which fits with the authors' conclusion that matrix remodeling participates in the vimentin function in collective migration. Please update the citation to the published version and, if appropriate, add one or two sentences in the Discussion to situate the MMP-dependent findings in light of MT1-MMP's specific role in vimentin-positive invasion.

Citation 17 has been updated to the published version. We have also a small paragraph about their MMP results in paragraph 4 of the discussion section.
